# Iminodibenzyl induced redirected COX-2 activity inhibits breast cancer progression

**DOI:** 10.1038/s41523-021-00330-9

**Published:** 2021-09-17

**Authors:** Harshit Shah, Lizhi Pang, Steven Qian, Venkatachalem Sathish

**Affiliations:** Department of Pharmaceutical Sciences, North Dakota State University, Fargo, ND USA

**Keywords:** Breast cancer, Drug development

## Abstract

Knocking down delta-5-desaturase (D5D) by siRNA or shRNA is a promising strategy to achieve 8-hydroxyoctanoic acid (8-HOA) production for cancer inhibition. However, the RNAi-based strategy to stimulate 8-HOA is restricted due to endonucleases mediated physiological degradation and off-target effects. Thus, to get persistent 8-HOA in the cancer cell, we recognized a D5D inhibitor Iminodibenzyl. Here, we have postulated that Iminodibenzyl, by inhibiting D5D activity, could shift the di-homo-gamma-linolenic acid (DGLA) peroxidation from arachidonic acid to 8-HOA in high COX-2 microenvironment of 4T1 and MDA-MB-231 breast cancer cells. We observed that Iminodibenzyl stimulated 8-HOA caused HDAC activity reduction resulting in intrinsic apoptosis pathway activation. Additionally, reduced filopodia and lamellipodia, and epithelial-mesenchymal transition markers give rise to decreased cancer cell migration. In the orthotopic breast cancer model, the combination of Iminodibenzyl and DGLA reduced tumor size. From in vitro and in vivo studies, we concluded that Iminodibenzyl could reprogram COX-2 induced DGLA peroxidation to produce anti-cancer activity.

## Introduction

Breast cancer is the most commonly occurring cancer among females in developed countries. As per the National Institute of Statistics, 90% of women are diagnosed with advanced-stage breast cancer, which significantly reduces their survival and quality of life^1^. Multiple predisposing factors are responsible for mammary cancer induction and growth, including overconsumption of ω-6 polyunsaturated fatty acid (ω-6-PUFAs)^2,3^. Scientific reports have suggested that cancer-promoting effects of ω-6-PUFAs are due to cyclooxygenase-2 (COX-2) induced increased levels of pro-carcinogenic eicosanoids, such as prostaglandin E_2_ (PGE_2_)^3^.

COX-2 is involved in the growth of various epithelial cancers including breast cancer^4^. An immunohistochemistry study evaluating more than 1500 breast cancer specimens for COX-2 reported moderate to strong COX-2 expression in 37% of the samples^5^. Additionally, a meta-analysis study, involving 6739 patients, demonstrated that higher levels of COX-2 in breast cancer are linked with poor prognosis, big tumor size, and lymph node metastasis^6^. A report by Ranger et al. also provided the correlation between COX-2 and distant metastasis by indirectly affecting mutagenesis, increased cell migration, angiogenesis, and apoptosis in breast cancer^7^. The overall relationship between COX-2 and PGE_2_, and tumor grade, tumor size, invasiveness, and survival in breast cancer has been established^5,8^. By considering the definitive role of COX-2, many COX-2 inhibitors were analyzed as a therapeutic modality against breast cancer and found to provide significant protection against breast cancer in various in vitro and murine animal studies^9,10^. However, the use of COX-2 inhibitors in clinical studies was controversial and inconclusive as long-term treatment was associated with prothrombic effects and cardiovascular adverse events. As a result of which, in 2005, the Drug Safety and Risk Management Advisory Committee barred the use of COX-2 inhibitors for cancer management^11^.

Recently, our lab has provided evidence about the implication of COX-2 (redirecting the use of COX-2 from inhibition to usage) to achieve cancer growth inhibition. ω-6-PUFAs like dihomo-γ-linolenic acid (DGLA) upon metabolism by delta-5-desaturase (D5D) generates arachidonic acid (AA), which further metabolizes by COX-2 in tumor cells to PGE_2_ causing tumor growth and metastasis_._ However, inhibition of the D5D enzyme causes building up of DGLA, which undergoes COX-2 stimulated peroxidation to yield a tumor growth-inhibiting agent 8-Hydroxyoctanoic acid (8-HOA). In our earlier studies, we have used D5D siRNA and shRNA to redirect COX-2 induced DGLA peroxidation to fuel 8-HOA production yielding significant protection against colon, pancreatic, lung, and breast cancer^12–17^. However, siRNA and shRNA approaches are linked with major limitations such as physiological degradation by endonucleases, off-target side effects, and inability to cross the cellular membrane due to the presence of negative charge on the phosphate backbone^18,19^. To overcome the limitations, we screened different reported D5D activity inhibitors^20–22^ and selected a more efficacious D5D inhibitor, Iminodibenzyl. This compound is a building block in widely-used antipsychotics (such as carbamazepine, carpipramine, and imipramine), which acts as an antagonist for alpha-1 and alpha-2 adrenoreceptors. However, the effect and mechanism of Iminodibenzyl on cancer have not been studied^20^.

Based on the preliminary studies and established D5D inhibition strategy, it was hypothesized that Iminodibenzyl can inhibit the DGLA metabolism to AA by inhibiting the D5D activity. The accumulated DGLA will be further peroxidized by overexpressed COX-2 to 8-HOA producing cancer growth inhibitory activity against breast cancer cells. To achieve the research goal, we have performed schematic studies to evaluate the biochemical change after Iminodibenzyl treatment, apoptosis analysis, and explored intrinsic apoptosis pathway, cell migration, epithelial-mesenchymal transition (EMT) markers, and performed an orthotopic breast cancer model to analyze in vivo activity.

## Results

### Iminodibenzyl induced altered DGLA metabolism resulted in 8-HOA production

**Fig. 1:**
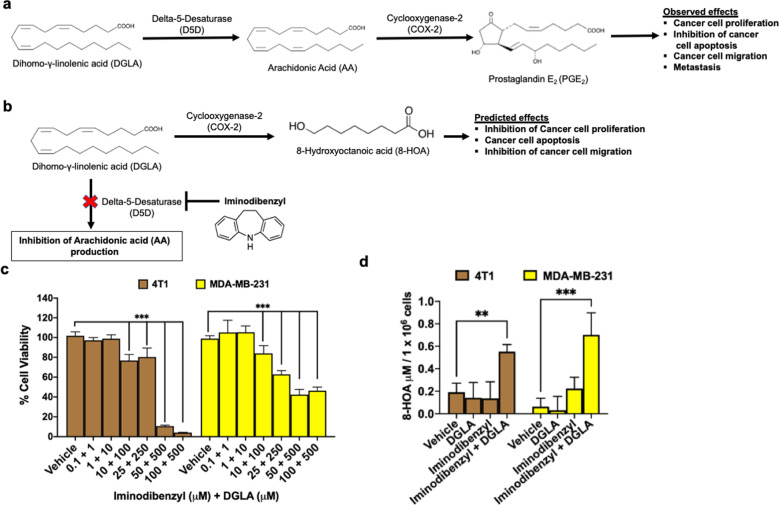
Redirecting COX-2 peroxidation through Iminodibenzyl in cancer management. **a** COX-2 induced ω-6 polyunsaturated fatty acid peroxidation leading to cancer progression and metastasis. **b** Hypothesized effect of Iminodibenzyl on COX-2 repurposing to produce 8-HOA from DGLA peroxidation resulting in cancer growth inhibitory effect. **c** Cell viability analysis by different dose combination of DGLA and Iminodibenzyl in 4T1 and MDA-MB-231 cancer cells. **d** GC/MS analysis of 4T1 and MDA-MB-231 cancer cells treated with 100 μM DGLA, 10 μM Iminodibenzyl, and combination of 100 μM DGLA + 10 μM Iminodibenzyl. *n* = 6–10 for (**c**) and *n* = 3 for (**d**). Data are represented as mean ± SEM. ***P < 0.001, **P < 0.01 vs. vehicle.

It is well established that ω-6-PUFAs such as DGLA upon metabolism by D5D forms AA, which further peroxidized by overexpressed COX-2 in cancer microenvironment to precancerous PGE_2_^23^. The generated PGE_2_ acts through various cellular pathways resulting in uncontrolled cancer cell proliferation, inhibition of cancer cell apoptosis, increased migration, and metastasis (Fig. 1a)^24^. On the contrary, D5D inhibition by Iminodibenzyl is hypothesized to inhibit DGLA metabolism to AA in breast cancer cells, causing DGLA deposition. The COX-2 in the tumor microenvironment peroxidize accumulated DGLA to a tumor growth-inhibiting metabolite 8-HOA, which is postulated to have an inhibitory effect on cancer cell proliferation, migration, and consequently induces apoptosis in the cancer cell (Fig. 1b).

In the initial analysis, the cell viability assay was performed to determine the suitable in vitro dose of DGLA and Iminodibenzyl for 4T1 and MDA-MB-231 breast cancer cells. When cells were treated with gradient doses of DGLA (1–500 µM) or Iminodibenzyl alone (0.1–50 µM) for 48 hours, no significant cytotoxic effect was observed on cancer cell lines except the highest employed dose of DGLA (500 µM) and Iminodibenzyl (50 µM) (Supplementary Fig. 1a and b). Surprisingly, when cells were provided combination treatment with Iminodibenzyl and DGLA, a significant reduction in cell viability was observed from Iminodibenzyl (10 µM) and DGLA (100 µM) in 4T1 and MDA-MB-231 cells (Fig. 1c, *P* *<* 0.001), implicating the benefit of the drug combination at lower dose. Hence, to achieve the ideal outcome and to avoid unexpected toxicity, we used 10 µM Iminodibenzyl + 100 µM DGLA in subsequent in vitro studies. To investigate the mechanism of the drug combination and feasibility of Iminodibenzyl as a D5D activity inhibitor in breast cancer cells, we quantified the 8-HOA level in breast cancer cells treated with 10 µM Iminodibenzyl + 100 µM DGLA. A significant increase in the 8-HOA level in COX-2 overexpressing 4T1 and MDA-MB-231 cancer cells was observed (Fig. 1d, *P* < 0.01 and <0.001, respectively). Notably, we did not find any significant change in 8-HOA level, when cells were provided treatment with Vehicle, DGLA, or Iminodibenzyl alone (Fig. 1d). To determine whether COX-2 is essential for our treatment efficacy, we assessed the cell viability in COX-2 negative non-cancerous breast epithelial cells (MCF-12a) by treating them with similar doses of DGLA (1–500 µM), Iminodibenzyl (0.1–50 µM), and a combination of them. Unlike cancer cells, no effect on cell viability was noted at the optimal concentrations (10 µM Iminodibenzyl and 100 µM DGLA, Fig. Supplementary Fig. 1c).

### Combination of Iminodibenzyl and DGLA caused breast cancer cell apoptosis and inhibition of migration

**Fig. 2:**
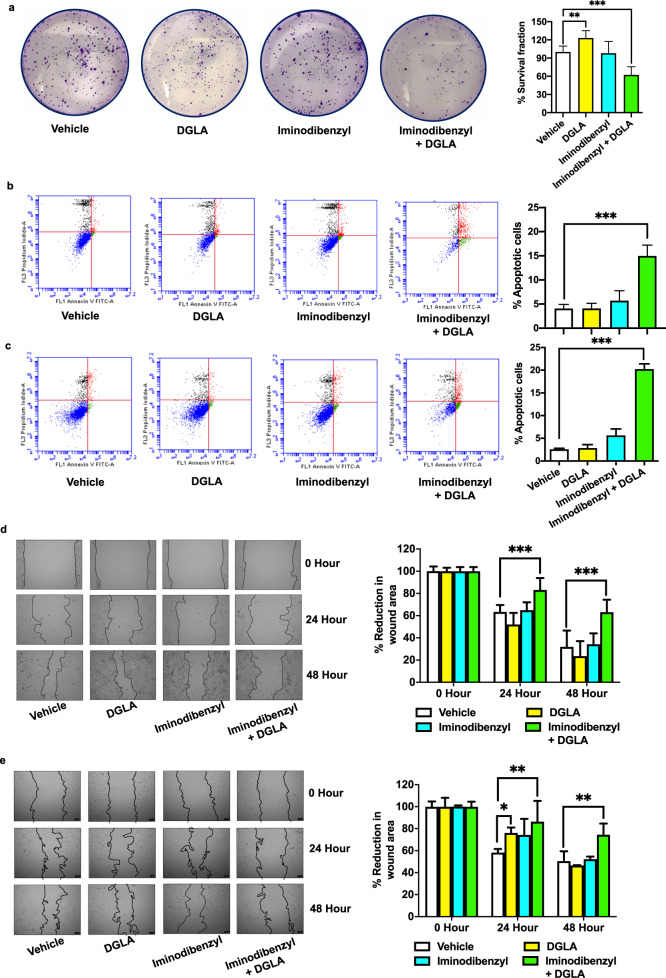
Iminodibenzyl induced DGLA peroxidation inhibited the in vitro 4T1 and MDA-MB-231 cancer cell proliferation and migration. **a** Proliferation analysis by colony formation assay in 4T1 cells and quantification of percentage survival fraction. **b** Apoptosis analysis in 4T1 cells by Annexin V-FITC/PI double staining and quantification of percentage apoptotic cells. **c** Apoptosis analysis in MDA-MB-231 cells by Annexin V-FITC/PI double staining and quantification of percentage apoptotic cells. **d** Wound-healing assay and quantification of the wound area (scale: 100 μm) at 0, 24, and 48 h in 4T1 cells. **e** Wound-healing assay and quantification of the wound area (scale: 100 μm) at 0, 24, and 48 h in MDA-MB-231 cells. In all the experiments 4T1 and MDA-MB-231 breast cancer cells were treated with Vehicle, 100 μM DGLA, 10 μM Iminodibenzyl, and combination of 100 μM DGLA + 10 μM Iminodibenzyl. *n* = 3–4 for (**a–e**). Data are represented as mean ± SEM. *P *<* 0.05, **P *<* 0.01, ***P *<* 0.001 vs. vehicle.

In our previous study, we observed a significant reduction in cancer cell proliferation and migration with D5D knockdown followed by DGLA treatment^12–15^. Since Iminodibenzyl diverted DGLA metabolism by D5D inhibition, we have performed a proliferation assay, apoptosis analysis, and migration assay to determine cancer progression opposing effects of the combination of Iminodibenzyl and DGLA. The colony formation assay was performed to assess the outcome of different treatments on cancer cell proliferation. From analysis, we found no prominent effect on cancer cell proliferation when 4T1 cancer cells were treated with Vehicle and 10 µM Iminodibenzyl. However, a significant decrease in the number of colonies was observed on providing concomitant treatment of Iminodibenzyl and DGLA (Fig. 2a, *P* *<* 0.001), which implicated a reduction in cancer cell proliferation. Interestingly, when cells were provided with 100 µM DGLA treatment, a significant increase in 4T1 cell proliferation was observed (Fig. 2a, *P* *<* 0.01*)*. Additionally in PI-Annexin V double staining apoptosis assay, we found a significant increase in the apoptotic positive rate of 4T1 and MDA-MB-231 cells treated with the combination of 100 µM DGLA and 10 µM Iminodibenzyl (Fig. 2b and c, *P* *<* 0.001). We did not observe any significant increase in the percentage of apoptotic cells when cells were provided DGLA or Iminodibenzyl. To further evaluate the effect of different treatments on cancer cell migration, we performed the wound-healing assay on 4T1 and MDA-MB-231 cells. We found a significant decline of cancer cell migration and larger wound size in 4T1, and MDA-MB-231 cancer cells at 24 h and 48 h of treatment of DGLA + Iminodibenzyl (Fig. 2d, *P* *<* 0.001, and Fig. 2e, *P* *<* 0.01*)*. We also performed the transwell assay to confirm the finding obtained from the wound-healing assay. Transwell migration assay also showed similar finding of reduced 4T1 cancer cell migration (larger bright spots) in the group of cells provided simultaneous treatment with 10 µM Iminodibenzyl and 100 µM DGLA (Supplementary Fig. 2, *P* *<* 0.01).

### Combination of Iminodibenzyl and DGLA induced activation of intrinsic apoptosis pathway

**Fig. 3:**
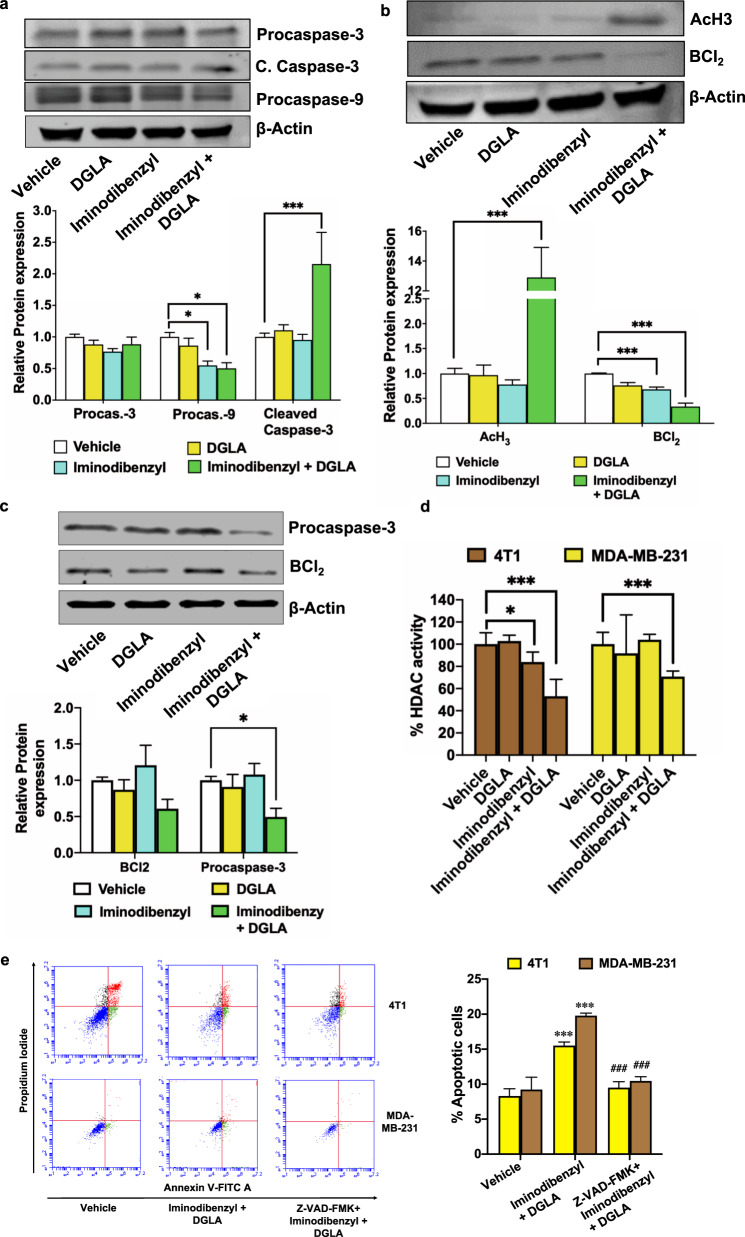
Activation of intrinsic apoptosis pathway after combination treatment with Iminodibenzyl and DGLA. **a**, **b** Western analysis and quantification of fold change in protein level (normalized to β-actin) involved in intrinsic apoptosis pathway (BCl_2_, procaspase-3, cleaved caspase-3, procaspase-9, AcH3) after 48 h treatment in 4T1 cells. **c** Immunoblot analysis and quantification of fold change in BCl_2_ and procaspase-3 after 48 h treatment in MDA-MB-231 cells. **d** Percentage HDAC activity analysis in 4T1 and MDA-MB-231 cancer cells. **e** Apoptosis analysis in 4T1 and MDA-MB-231 cells treated with 30 μM Z-VAD-FMK for two hours prior DGLA and Iminodibenzyl combination treatment by Annexin V-FITC/PI double staining and quantification of percentage apoptotic cells. *n* = 3 for (**a**–**e**). Data are represented as mean ± SEM. ***P < 0.001, *P *<* 0.05 *vs*. vehicle, ^*###*^*P* *<* 0.001 vs. Iminodibenzyl + DGLA.

In our earlier studies, we have seen that D5D silencing by RNAi therapy caused caspase dependent death mechanism activation^12–16^. Additionally, PI-Annexin apoptosis analysis also showed an increased percentage of apoptotic cells by Iminodibenzyl and DGLA combination treatment (Fig. 2b). Hence, to find the plausible mechanism behind cell apoptosis, we analyzed procaspase-3, procaspase-9, and cleaved caspase-3 by western and microscopic analysis. The analysis showed a significant decrease in procaspase-9 level with 10 µM Iminodibenzyl, and the combination of 10 µM Iminodibenzyl and 100 µM DGLA treatment (Fig. 3a, *P* *<* 0.05). Consequently, cleaved caspase-3 (C. Caspase-3) analysis by western and immunofluorescence showed a significant increase in its levels on providing simultaneous treatment with 10 µM Iminodibenzyl and 100 µM DGLA in 4T1 cancer cells (Fig. 3a and Supplementary Fig. 3, *P* *<* 0.001). However, we did not observe any modulatory effect on apoptosis markers by DGLA treatment (Fig. 3a). Additionally, a significant reduction in anti-apoptotic protein BCl_2_ expression and significant increase in AcH_3_ was found with Iminodibenzyl alone and/or in combination with DGLA in 4T1 (Fig. 3b, *P* *<* 0.001). We did not observe any modulatory effect in DGLA or Iminodibenzyl alone treated MDA-MB-231 cells (Fig. 3c). Previously, we have demonstrated that exogenous 8-HOA or DGLA-derived 8-HOA could inhibit HDAC activity in cancer cells^13–15^. We hypothesized that the combination of Iminodibenzyl and DGLA would also have a dampening effect on HDAC activity via endogenous 8-HOA in breast cancer cells. We observed a significant reduction of HDAC activity in 4T1 and MDA-MB-231 cells treated with Iminodibenzyl and DGLA combination (Fig. 3d, *P* *<* 0.001). We also noted a significant reduction in HDAC activity in 4T1 cells treated with 10 µM Iminodibenzyl alone (Fig. 3d, *P* *<* 0.05). To validate the caspase dependent apoptotic mechanisms, we treated cells with 30 µM Z-VAD-FMK (caspase inhibitor) for 2 h before providing cells the combination treatment and performed apoptosis analysis. We observed a significant increase in the percentage of apoptotic 4T1 and MDA-MB-231 cells (PI-Annexin V analysis) after Iminodibenzyl and DGLA combination treatment. Whereas, pretreatment with Z-VAD-FMK recuperated the percentage of apoptotic cells to the vehicle-treated cells (Fig. 3e, *P* *<* 0.001). Additionally, we also performed polarity-sensitive indicator of viability and apoptosis (pSIVA) imaging analysis. In line with the PI-Annexin analysis, we found that DGLA and Iminodibenzyl could result in a significant increase in the percentage of apoptotic breast cancer cells. Here, the pretreatment of Z-VAD-FMK diminished the DGLA and Iminodibenzyl induced apoptosis in pSIVA imaging (Supplementary Fig. 5, *P* *<* 0.001).

### Iminodibenzyl induced DGLA peroxidation modulates cell migratory and EMT markers

**Fig. 4:**
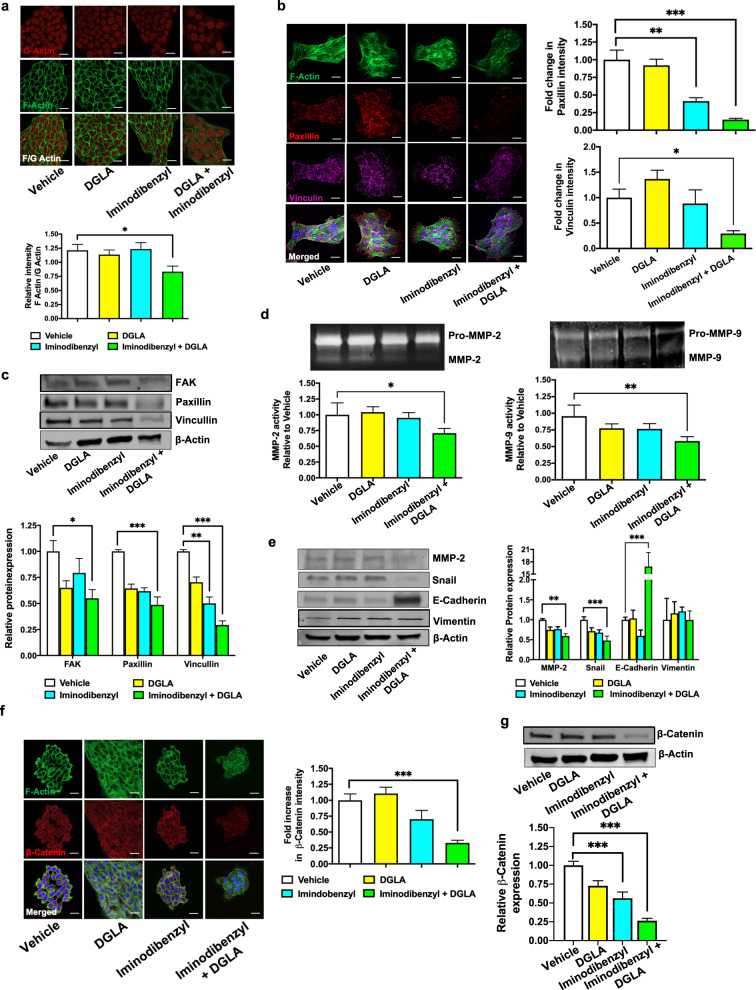
Iminodibenzyl and DGLA combination inhibited the migration and metastasis markers in 4T1 cancer cells. **a** Immunofluorescence analysis and quantification of F/G actin level (scale: 20 μm). **b** Immunofluorescence analysis and quantification of fold change in protein intensity for vinculin and paxillin in cancer cells by confocal microscope (scale: 20 μm). **c** Western analysis and quantification of fold change in protein level (normalized to β-Actin) involved in 4T1 breast cancer cell migration (FAK, Paxillin, Vinculin) after 48 h treatment. **d** In vitro gelatin gel zymography for MMPs released in the cell culture medium. **e** Western analysis and quantification of fold change in protein level (normalized to β-Actin) involved metastasis (MMP-2, Snail, E-Cadherin, and Vimentin). **f** Immunofluorescence analysis for β-Catenin in 4T1 breast cancer cells (Scale: 20 μm). **g** Western analysis and quantification of fold change in β-Catenin (normalized to β-Actin) after 48 h treatment in 4T1. *n* = 3 for (**a**–**g**). Data are represented as mean ± SEM. ***P < 0.001, **P *<* 0.01, *P *<* 0.05 vs. vehicle.

To identify the mechanistic role of Iminodibenzyl and DGLA on reduced cancer cell migration, we analyzed the relative F-Actin level by the Immunocytochemistry. Significant reduction in F-Actin/G-Actin (crucial protein in filopodia and lamellipodia, which are essential for cell motility) ratio was observed when 4T1 breast cancer cells were provided concomitant treatment with DGLA and Iminodibenzyl (Fig. 4a, *P* *<* 0.05). Further, we analyzed the Paxillin and Vinculin proteins, which are prime elements involved in filopodia and lamellipodia. Western analysis showed a significant decline in Paxillin and Vinculin levels with combination treatment (Fig. 4c, *P* *<* 0.001), and this was further confirmed by immunofluorescence analysis, where a significant reduction in Paxillin and Vinculin was observed (Fig. 4b, *P* *<* 0.001 and *P* *<* 0.05, respectively). Notably, Iminodibenzyl alone treatment also showed significant depletion of Paxillin (Fig. 4b, *P* *<* 0.01) and Vinculin (Fig. 4c, *P* *<* 0.01). Additionally, we also found reduced focal adhesion kinase (FAK) with the combination treatment (Fig. 4c, *P* *<* 0.05). EMT is another important phenomenon to consider during cancer therapeutic analysis. Changes in EMT markers such as matrix metalloproteinases 2 and 9 (MMP-2 and MMP-9), Vimentin, Snail, and E-Cadherin lead to the displacement of the cancer cell to the systemic environment, which further localized at distant places to form metastatic nodules. From the zymography study, a significant reduction in MMP-2 and MMP-9 activity was noticed in 4T1 cells treated with concomitant supplementation of 10 µM Iminodibenzyl and 100 µM DGLA (Fig. 4d, *P* *<* 0.05 *and P* *<* 0.01). However, the protein expression of vimentin was consistent among all the treatment groups (Fig. 4e). A combination of Iminodibenzyl and DGLA significantly upregulated E-cadherin (Fig. 4e, *P* *<* 0.001) and down regulated MMP-2 (Fig. 4e, *P* *<* 0.01) and Snail (Fig. 4e, *P* *<* 0.001) level in 4T1 (Fig. 4e, *P* *<* 0.001). Additionally, we observed a significant decrease in MMP-2 and increase in E-cadherin expression when MDA-MB-231 cells were treated with the combination of Iminodibenzyl and DGLA (Supplementary Fig. 6, *P* *<* 0.001 and *P* *<* 0.01 respectively). Further, we observed significantly reduced β-catenin protein expression in 4T1 (Fig. 4g and f, *P* *<* 0.001) cells after treatment with Iminodibenzyl and DGLA. It is also important to note that, we also observed significantly diminished β-catenin levels in Iminodibenzyl alone treated 4T1 cells (Fig. 4g, *P* *<* 0.001).

### In vivo cancer growth inhibition by Iminodibenzyl fueled paradigm shift of COX-2 induced DGLA peroxidation

**Fig. 5:**
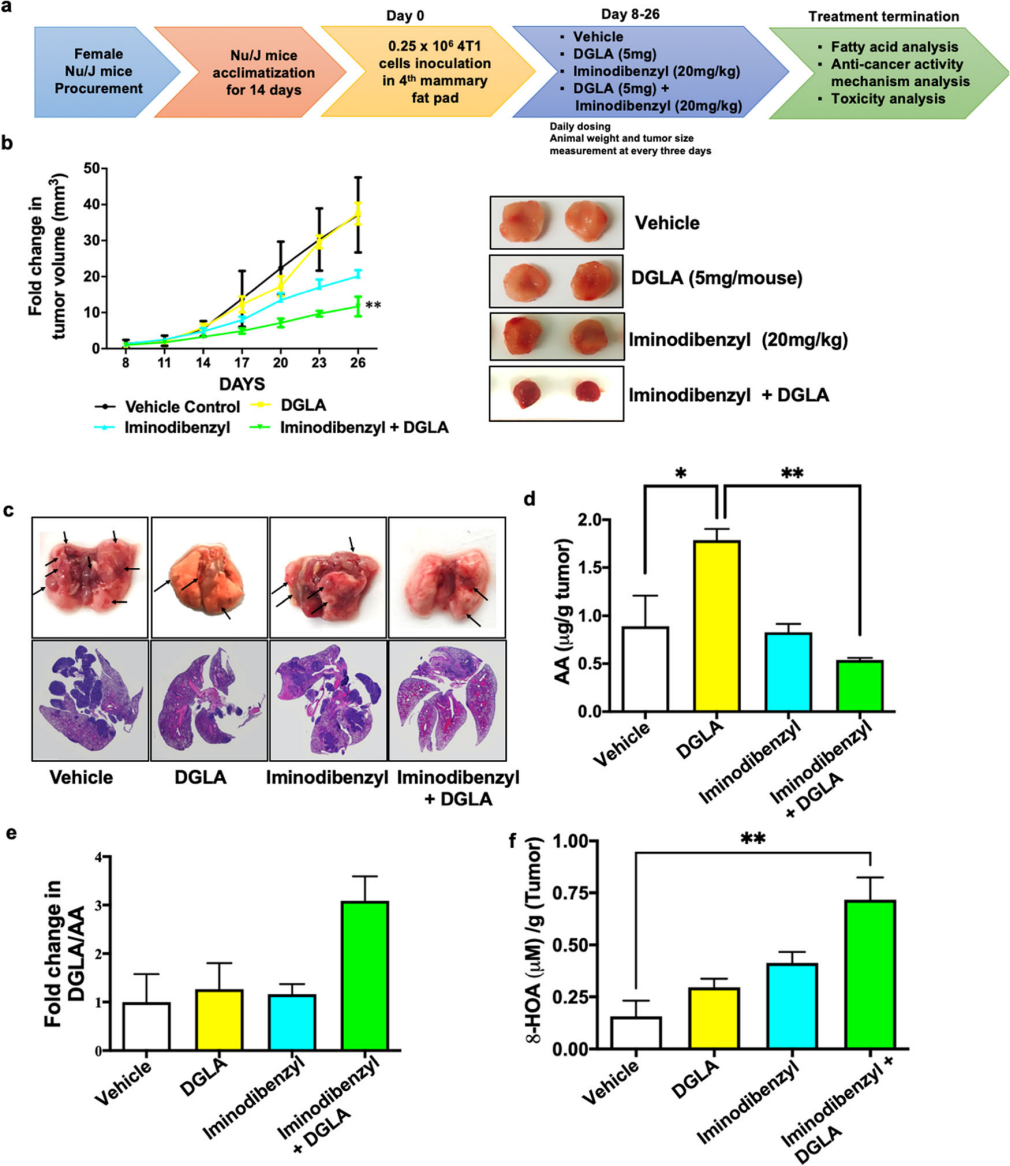
Iminodibenzyl stimulated in vivo DGLA peroxidation resulting in reduced cancer growth and lung metastasis nodules. **a** Schematic representation of the animal study plan. **b** Fold change in tumor volume and representative images of the tumor harvested from animals treated with Vehicle, DGLA (5 mg), Iminodibenzyl (20 mg/kg), and DGLA (5 mg) + Iminodibenzyl (20 mg/kg) treatments. **c** Representative images of the whole lung, and Hematoxylin and Eosin staining of lung showing lung metastatic nodules. **d** Arachidonic acid (AA) measurement by LC/MS, extracted by solid-phase extraction, from tumors obtained from animals treated with Vehicle, DGLA (5 mg), Iminodibenzyl 20 mg/kg, and DGLA (5 mg) + Iminodibenzyl (20 mg/kg). **e** LC/MS determination of fold change in DGLA/AA from tumors harvested from different groups of animals. **f** 8-HOA determination by GC/MS from tumor samples obtained from different treatment groups of animals. *n* = 6 for (**b**, **c**) and n = 3 for (**d**–**f**). Data are represented as mean ± SEM. **P *<* 0.01, *P *<* 0.05 vs. vehicle.

To analyze in vivo therapeutic effect of various treatment groups, we developed an orthotopic breast tumor model by inoculating 4T1 cells in the mammary fat pad of the female Nu/J mice. After eight days of cancer cell implantation, animals were randomly divided into four treatment groups (Vehicle, DGLA (5 mg), Iminodibenzyl (20 mg/kg), and Iminodibenzyl (20 mg/kg) + DGLA (5 mg)) having six animals in each group (Fig. 5a), and provided daily treatment. From the tumor volume measurement and visual inspection of the tumors at the end of the study, we noticed a significant decline in tumor volume in the group of animals receiving a combination of Iminodibenzyl (20 mg/kg) and DGLA (5 mg) as a treatment, while animals in Vehicle and DGLA treatment groups did not exert any significant effect on the tumor growth (Fig. 5b, *P* *<* 0.01 and Fig. 5c). It is important to note that animals in the Iminodibenzyl treatment group exhibited a moderate reduction in tumor volume (Fig. 5b and c). Additionally, from visual inspection, and hematoxylin and eosin (H&E) staining investigation of the lungs, we detected significantly less number (*n* = 6) of metastatic lung nodules in the group of animals that received simultaneous treatment of Iminodibenzyl and DGLA compared to animals administered Vehicle (*n* = 17), DGLA (*n* = 9), or Iminodibenzyl (*n* = 8) (Fig. 5c). From fatty acid analysis, we observed a significant increase in AA in tumors obtained from animals treated with DGLA alone (Fig. 5d; *P* *<* 0.05), which may be due to COX-2 induced DGLA peroxidation. However, animals from combination treatment groups did not show a significant increase in AA content (vs. vehicle). Contrary, animals administered Iminodibenzyl along with DGLA showed significantly reduced AA content (*vs*. DGLA treatment group), which could be due to the inhibited D5D activity by Iminodibenzyl causing diversion of DGLA metabolism (Fig. 5d, *P* *<* 0.01). This finding was further supported by increased DGLA and DGLA/AA levels in animals provided DGLA alone or in combination (Supplementary Fig. 7a and Fig. 5e). Consequently, 8-HOA level determination from tumor samples showed a significant increase in 8-HOA level when animals were provided the combination of Iminodibenzyl and DGLA as a treatment (Fig. 5f, *P* *<* 0.01). Additionally, we also performed pharmacokinetic (PK) analysis to find the bioavailability. Based on PK analysis, we quantified approximately 6-9% of total administered Iminodibenzyl reached the site of action (Supplementary Fig. 7b and 7c, *P* *<* 0.001).

**Fig. 6:**
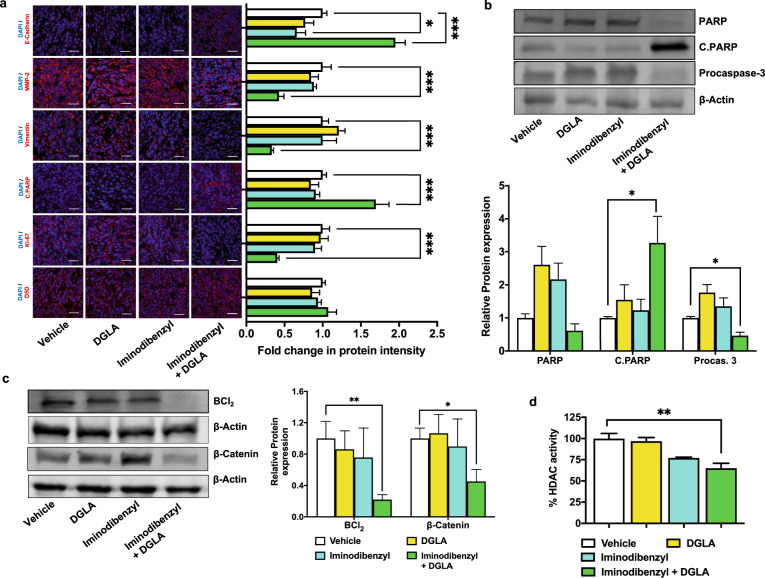
In vivo modulating effect of combination of Iminodibenzyl and DGLA on cancer growth and metastasis marker. **a** Immunohistochemical analysis of tumor samples for E-cadherin, MMP-2, Vimentin, C. PARP, Ki-67, and D5D (scale: 20 μm). **b** Western analysis and quantification of fold change (normalized to β-Actin) in apoptosis marker markers (PARP, C. PARP, and procaspase-3) from tumor samples extracted from animals treated with Vehicle, DGLA (5 mg), Iminodibenzyl 20 mg/kg, and DGLA (5 mg) + Iminodibenzyl (20 mg/kg). **c** Western analysis and quantification of fold change (normalized to β-Actin) in BCl_2_, and β-Catenin from tumor samples obtained from animals from different treatment groups. **d** Percentage HDAC activity from the tumor samples harvested from animals treated with Vehicle, DGLA (5 mg), Iminodibenzyl (20 mg/kg), and DGLA (5 mg) + Iminodibenzyl (20 mg/kg). n = 3 for (**a**–**d**). Data are represented as mean ± SEM. ***P < 0.001, **P *<* 0.01, *P *<* 0.05 vs. vehicle.

To elucidate the probable in vivo mechanism of action behind reduced tumor size and metastatic lung nodule, we analyzed the relative expression of different markers involved in apoptosis and EMT. Immunohistochemistry analysis showed a significant increase in E-Cadherin, and reduction in MMP-2 and Vimentin level in tumor samples obtained from animals simultaneously administered Iminodibenzyl (20 mg/kg) and DGLA (5 mg) (Fig. 6a, *P* *<* 0.001). We did not find any significant change in any analyzed EMT markers (MMP-2, Vimentin, and E-Cadherin) in tumors extracted from animals treated with Vehicle, DGLA, or Iminodibenzyl alone (Fig. 6a). Moreover, a significant reduction in proliferative marker Ki-67 was found in tumor samples from animals provided combination treatment (Fig. 6a, *P* *<* 0.001). D5D protein level analysis by Immunohistochemistry (Fig. 6a) and western (data not shown) showed no significant change in protein levels with any of the provided treatments. C.PARP expression further showed a significant increase in fluorescent signal with concurrent treatment with Iminodibenzyl and DGLA (Fig. 6a, *P* *<* 0.001). This finding was further supported by increased relative C. PARP expression (Fig. 6b, *P* *<* 0.05). Procaspase-3 and an anti-apoptotic protein BCl_2_ analysis in tumors showed similar findings as that of in vitro analysis, which was a significant reduction in the procaspase-3, BCl_2_, and β-Catenin protein levels in tumors harvested from the combination treatment of Iminodibenzyl and DGLA (Fig. 6b, *P* *<* 0.05 *for* procaspase-3 and Fig. 6c*, P* *<* 0.01 *for* BCl_2_ and *P* *<* 0.05 *for* β-Catenin). However, we did not see any significant changes in the PARP protein level in animals treated with Iminodibenzyl and DGLA alone compared to vehicle. HDAC activity analysis of protein samples extracted from Iminodibenzyl and DGLA treated animals showed a significant reduction in HDAC activity (Fig. 6d, *P* *<* 0.01). We also detected a notable reduction in HDAC activity in tumor samples obtained from animals treated with Iminodibenzyl alone with no significant changes in other treatment groups.

## Discussion

As per the American Cancer Society, breast cancer is the most frequently identified cancer in the female population. The estimated number of cases is increased by 30% (~284,000) in 2021^25^. Despite the therapeutic advancement and multiple therapeutic modalities, breast cancer will be responsible for more than 40,000 deaths in women in 2021^25^. In cancer cells, ω-6-PUFAs (like DGLA) were concerted to downstream metabolites like AA by the enzyme D5D. The produced AA is further acted upon by COX-2 to produce PGE_2_, which acts in an autocrine and paracrine manner through various molecular pathways causing breast cancer growth, invasion, and metastasis^26,27^. Multiple reports have suggested the role of inducible COX-2 as a ubiquitous driver for breast cancer development^23,28–31^. By studying the role of COX-2 in tumor development, clinical trials were conducted evaluating selective and non-selective COX-2 inhibitors for various cancer management. However, COX inhibitors alone or in combination with other chemotherapeutic agents failed to protect against cancer, and contrary, they produced cardiovascular adverse events resulting in the death of clinical subjects^11,29,32–34^.

Recently, our lab has demonstrated the COX-2 induced DGLA peroxidation in D5D knockdown (siRNA/shRNA/3-way junctional (3WJ) D5D siRNA nanoparticle) cells and resulting in cancer growth inhibition against colon, pancreatic, lung, and breast cancer by 8-HOA production^12–17^. We have also found that when 8-HOA was administered with other chemotherapeutic agents like gemcitabine or 5-fluorouracil, a significant synergistic effect was noted causing significant inhibition of cancer cell proliferation and apoptosis^14,15^. However, the exogenous administration of 8-HOA and D5D inhibition by siRNA or shRNA-based approach to producing 8-HOA is restricted by off-target side effects, endonucleases mediated physiological destruction, and the inability of RNAi molecules to cross biological membrane^19^. To overcome the limitations of the siRNA-based delivery system and to achieve sustained in vivo 8-HOA production, we examined different reported D5D activity inhibitors, including Sesame^21^, Iminodibenzyl^20,22^, CP-24879^35^, and Curcumin^36^. Among all identified D5D inhibitors, Iminodibenzyl has been found with the highest efficacy (IC_50_: 104 nM) to inhibit D5D activity followed by CP-24879 (144 nM), curcumin (148 nM), and Sesame (346 nM) in the rat liver microsome system. Moreover, only Iminodibenzyl was able to produce 8-HOA above the threshold level (>0.5 µM), resulting in a significant reduction in survival fraction in colon cancer cells^20^. Accordingly, in the current study, we investigated the potential role of Iminodibenzyl in the breast cancer management.

As per the previously published reports, D5D silencing in COX-2 overexpressed cells redirect DGLA metabolism to 8-HOA and simultaneously produce less AA. Iminodibenzyl halts the D5D activity resulting in less AA and increased DGLA/AA ratio in breast cancer. The accumulated DGLA was then metabolized by COX-2 in breast cancer cells to 8-HOA. The produced 8-HOA may lead to decreased cell viability in 4T1 and MDA-MB-231 cells. However, the combination of Iminodibenzyl and DGLA not affecting the cell viability of epithelial MCF-12a cells, possibly due to negative COX-2 expression. This phenomenon emphasizes the importance and role of 8-HOA in the DGLA + Iminodibenzyl combination in cancer cells. Further, no effect on MCF-12a cell viability and minuscule 8-HOA production further supported the COX-2 dependent mechanism of Iminodibenzyl. Notably, higher concentrations (500 µM DGLA alone or with Iminodibenzyl) showing some toxic effect on cells, possibly due to other effects. For example, known effect by ω-6-PUFAs like linoleic acid (LA) and gamma-linolenic acid (GLA) at high concentrations have been found to cause significant reduction in cell viability by inducing reactive oxygen or carbonyl species formation and/or activating apoptosis by various mechanisms including caspase dependent mechanism^37,38^.

In line with the previously published findings of increased apoptosis after providing DGLA as a treatment in D5D knocked down cancer cells^13^, we also observed increased apoptosis upon a combination of Iminodibenzyl and DGLA treatment in both used cancer cell lines. Additionally, a significant reduction in tumor volume was observed when female mice were provided concurrent treatment of Iminodibenzyl and DGLA in the orthotopic breast cancer study. During apoptosis, the intrinsic death pathway regulated by BCl_2_ is evoked, which activates the caspase cascade initiator procaspase-9 followed by procaspase-3. The activated procaspase-3 then fragmented to cleaved caspase-3 to induce apoptosis^39–41^. The combination treatment of Iminodibenzyl and DGLA activated the intrinsic apoptosis death mechanism in in vitro and in vivo, confirmed using pan-caspase inhibitor Z-VAD-FMK.

Abnormal histone acetylation has an essential role in tumor development by epigenetically inhibiting the tumor suppressor genes, and hence histone deacetylase inhibition has become the ideal target for cancer therapeutics^42^. HDAC inhibitors exhibit their anti-cancer activity by stimulating acetylation of core histone protein, which impacts gene transcription resulting in apoptosis and degradation of misfolded proteins^43^. Iminodibenzyl administration redirected the DGLA metabolism to 8-HOA, which also exerted a significant reduction in HDAC activity^22^. The reduced HDAC activity linked with upregulation of AcH_3_ (Supplementary Fig. 4), which could activate the intrinsic apoptosis pathway to achieve tumor cell apoptosis and reduced cell proliferation ensuing in reduced tumor size.

During metastasis, the tumor cell migrates through the extracellular matrix (ECM) for intravasation into lymphatic and blood vessels. This is achieved by various types of movement like chemoattractant mediated chemotaxis and environmental gradient mediated haptotaxis^44^. Chemotaxis is one of the mechanisms through which cancer cells migrate. Cancer cell migration is a complex mechanism that necessitates coordination between specific cellular processes such as aggregation and dispersal of focal adhesions (FAs), polymerization of the actin filament, etc.^45^ The FA formation is commenced by the interaction of integrins with corresponding ligands followed by recruitment of various proteins such as focal adhesion kinase (FAK), vinculin, and paxillin^45^. Additionally, actin filament, specifically F-actin is also involved in processes like filopodia, lamellipodia, and invadopodia, which help cancer cells to move and invade distant sites to colonize^46^. Reduced FAK, paxillin, and vinculin levels supported the findings of reduced filopodia and lamellipodia in 4T1 breast cancer cells, which might have resulted in reduced cancer cell migration as visible from large wound size in wound-healing assay. During cancer metastasis, EMT is an important complex phenomenon leading to metastatic nodule formation at the distant site. It is mediated by intricate crosstalk between tumor cells and adjacent stroma and cannot be fully simulated in vitro^47^. Growth factors like TGF-β are widely used in vitro models to stimulate mesenchymal phenotype from epithelial cells (MCF-7a, MCF-12a, etc.)^48,49^. Interestingly, TGF-β exposure not be able to stimulate the COX-2 expression in the breast cancer cells^50^, and this was further confirmed by our own (data not shown). Accordingly, TGF-β induced EMT model seems not appropriate in our proposed strategy. Breast tumor cells excrete zinc-dependent proteases such as MMP-2 and MMP-9, which damage an integral part of the basal membrane, type IV collagen, resulting in cancer cells’ entry into the systemic circulation. Iminodibenzyl along with DGLA administration caused a differential reduction in MMP-9 and MMP-2 activity and expression inferring reduced ability to invade, indicating a possible independent effect on MMPs expression and activity. Furthermore, increased E-Cadherin, a single-span transmembrane protein essential for tight inter-epithelial cell connections, was showing improved protection against metastasis ability of the 4T1 and MDA-MB-231 cancer cells. Snail and Vimentin are other markers responsible for tumor recurrence and tumor cell metastasis^51^, which was found to be significantly reduced with the Iminodibenzyl and DGLA combination treatment. We believe that the reduced cancer cell migration and downregulated protein markers involved in EMT caused decreased metastatic lung nodules in the orthotopic breast cancer model.

Several studies have demonstrated the relation between the canonical Wnt/β-catenin pathway and upregulated proliferation, survival, and metastasis by modulating the intrinsic apoptosis pathway and EMT in breast cancer^52–56^. β-catenin is an appealing target due to its ability to abolish cancer relapse^57^. From in vitro and in vivo analysis, we have perceived that Iminodibenzyl and DGLA combination caused downregulation of β-catenin, which could be another possible mechanism leading to breast cancer cell apoptosis and reduced cell proliferation leading to reduced tumor growth by Iminodibenzyl and DGLA combination treatment. The downregulated β-catenin also has an inhibitory role in EMT resulting in less metastasis of tumor cells from the site of inoculation to the lungs in the orthotopic tumor model.

From all the above in vitro and in vivo findings, we summarize that Iminodibenzyl being a potent D5D activity inhibitor, diverting COX-2 induced DGLA metabolism to cancer demolishing 8-HOA. The produced 8-HOA inhibits HDAC activity and simultaneously activates the intrinsic apoptosis pathway causing tumor growth inhibition. Additionally, combination treatment downregulated the β-catenin level, which subsequently reduced proteins involved in filopodia and lamellipodia causing reduced cancer cell migration. In nutshell, Iminodibenzyl along with the DGLA administration strongly holds the promise to provide therapeutic protection against breast cancer growth.

## Methods

### Cell lines

A COX-2 overexpressing triple-negative murine breast cancer cell line 4T1, Human breast cancer cell line MDA-MB-231, and a non-cancerous human epithelial cell line MCF-12a were obtained from American Type Culture Collection (ATCC). All the cell lines were cultured according to ATCC standard culture protocol.

### In vitro dose determination study for DGLA, Iminodibenzyl, and combination of DGLA and Iminodibenzyl

MTT (3-(4,5-dimethyl-2-thiazolyl)-2,5-diphenyl-2H-tetrazolium bromide; Alfa Aesar; catalog number: L11939 (MA, USA)) assay was used to determine the dose to be used for in vitro study. Approximately 5 × 10^3^ 4T1, MDA-MB-231, and MCF-12a cells (in 100 μL medium) were seeded in a 96-well culture plate. After overnight incubation, cells were treated with DGLA (1–500 μM), Iminodibenzyl (10,11-dihydro-5H-dibenz[b,f]azepine, Sigma (MO, USA), Catalog number: I1308, CAS Number: 494-19-9) (0.1–50 μM), and combination of Iminodibenzyl and DGLA (1:10 ratio from 0.1 μM Iminodibenzyl + 1 μM DGLA to 50 μM Iminodibenzyl + 500 μM DGLA) in dimethyl sulfoxide (DMSO) for 48 hours. At the end of the treatment, MTT (5 mg/ml) was added to the cells. After 3 h of incubation, the formed formazan crystals were dissolved by adding DMSO, and absorbance was measured at 570/650 nm^16^.

### Colony formation assay

To determine the anti-proliferative effect of different treatment modalities, a colony formation assay was performed. For this assay, approximately 1 × 10^3^ 4T1 cells were seeded in the 6-well culture plates and different treatments: Vehicle, 100 μM DGLA, 10 μM Iminodibenzyl, and a combination of 10 μM Iminodibenzyl and 100 μM DGLA were provided for 48 h. After treatment, cells were washed and incubated with a fresh DMEM growth media until the colonies were formed. The colonies were then fixed and stained by using 0.5% W/V crystal violet solution. After staining, images were taken and analysis was done by ImageJ software^12,13^.

### Apoptosis analysis

The apoptosis analysis was done using PI-Annexin V double staining method as per the standard procedure (BD Pharmingen, Catalog number: 556547). Briefly, the cells were provided Vehicle, DGLA, Iminodibenzyl, and combination. To validate caspase dependent apoptosis mechanism, cells were pretreated with 30 μM Z-VAD-FMK (Santacruz biotechnology, Catalog number: SC3067) for 2 h, followed by Iminodibenzyl and DGLA treatment. For apoptosis analysis, the cells were collected by trypsinization. The cells were washed twice with ice-cold PBS buffer, followed by mixed with 100 μl 1× binding buffer at a concentration of 1 × 10^6^ cells/ml. The cells were then stained with 5 μl PI and 5 μl Annexin V FITC for 30 min in dark at room temperature. After incubation, the volume was made till 500 μl and the reading was taken by Acuri C6 flow cytometer. The data analysis was done by Acuri C6 software as described elsewhere^12,13^.

### Wound-healing assay

The wound-healing assay was conducted to determine the alleviating effect of treatments on 4T1 and MDA-MB-231 cell migration. To perform the wound-healing assay, approximately 0.25 × 10^6^ 4T1 cells were seeded in a 6-well culture plate and allowed to form a monolayer. After that, the wound was made by gently scratching the cell monolayer with a sterile pipette tip. The cells were washed 2–3 times to remove any dislodged cells and followed the treatments as above. The wound size was captured at 0, 24, and 48 h by brightfield microscopy using a Leica Microsystem. The wound area at different time points was measured by using ImageJ software and the reduction in wound area was calculated and analysis was done^12,13^.

### Transwell assay

The assay was executed by using the Costar transwell chamber (6.5 mm insert, 8.0 μm polycarbonate membrane). To perform the transwell assay, approximately, 25 × 10^3^ 4T1 cells were seeded in the insert. The next day, the media was replaced with serum-free DMEM media containing different treatments for 48 h. After treatment, the cells were fixed by PFA and stained using crystal violet. The cells on the upper side of the chamber were scraped off by a cotton stick and the images of the migrated cells across the membrane were taken by Lecia Microsystems. The migration rate was determined by using ImageJ as the area covered by the cell after the treatment^16,17^.

### Zymography

To perform the gelatin gel zymography, 4T1 cells were seeded and treatments were provided for 48 h. After treatment, the conditioned media from different treatment groups was collected and concentrated using Vivaspin 6 MWCO 3000 concentrator spin columns (28-932293; GE Healthcare, Waukesha, WI, USA). The protein content in each group was analyzed using a BCA protein assay kit (ThermoFisher Scientific, catalog number: 23225). The concentrated media (~20 μg protein) was then diluted with Bio-Rad Zymogram sample buffer (Catalog number: 161-0764) and loaded on Novex 10% Zymogram Plus (Gelatin) Protein Gels (ThermoFisher Scientific, Catalog number: ZY00100 Box). After electrophoresis, the zymography gel was incubated on a shaker in zymogram renaturation buffer (Bio-Rad, catalog number: 161-0765) for one hour. Then, the gel was washed with deionized water and incubated for 36 h in a zymogram develop buffer (Bio-Rad, catalog number: 161-0766) at 37° C. The gelatin gel was then stained with Coomassie Blue R-250 staining solution (Bio-Rad, catalog number: 1610436) for 2 h at room temperature and then destained using a solution (10% glacial acetic acid, 40% methanol, and 50% deionized water). The gels were then imaged by the Li-Cor Odyssey XL System and MMP activity was analyzed^58^.

### Orthotopic breast cancer model

The orthotopic mammary cancer model was established by using six weeks old female homozygous NU/J mice from The Jackson Lab (Sacramento, USA). The protocol for the orthotopic breast cancer model was prior sanctioned by the Institutional Animal Care and Use Committees (IACUC) at North Dakota State University (NDSU). After two weeks of acclimatization, breast cancer was induced by injecting 4T1 tumor cells into the fourth mammary fat pad, and animals were housed for additional seven days for the tumor to grow^17^. Then, the mice were randomized into four treatment groups having six mice in each group. During the study, animals were dosed daily (Vehicle, DGLA (Cayman chemicals, MI, USA) (5 mg/mouse, p.o.), Iminodibenzyl (20 mg/kg, i.p.), and a combination of Iminodibenzyl ((20 mg/kg, i.p.) + DGLA (5 mg/mouse, p.o.)). During the treatment period, the change in animal weight and the tumor volume were noted every three days. Tumor volume was measured using a digital Vernier caliper ((V = L × W^2^)/2 (L: longest axis; W: shortest axis)). At the end of the study, blood, tumors, and vital organs (liver, spleen, heart, and kidneys) from animals were collected and analysis was done.

### 8-HOA quantification

To determine 8-HOA from the cell, 1 ml of 4T1 cell suspension was taken from different treatment groups. For tumor samples, a small piece of tumor was taken, immersed in liquid nitrogen, and ground into very fine powder. One ml of cell suspension and tumor solution (in HPLC grade water) was mixed with 500 μl methanol containing hexanoic acid as internal standard, 50 μl 1 N HCl, and 3 ml dichloromethane. The sample solution was then vortexed, centrifuged, and the organic layer was separated. This step was repeated twice. The obtained organic layer was dried in a vacufuge. The samples were then reconstituted in diisopropylethylamine (50 μl, 1.0% v/v) and PFB-bromide (Pentaflurobenzyl-bromide) in acetonitrile (1.0% v/v) for 30 min at 37° C. Then, 2 μl of the sample was injected into a gas chromatograph (Agilent 7890A). The peaks of Hexanoic acid and PFB derivatized 8-HOA were analyzed at extracted ion current with *m/z* 181. The concentration of 8-HOA was determined by extrapolating the value of the ratio of the area of the 8-HOA peak and hexanoic acid peak into the internal standard curve^12,13,16,17^.

### DGLA and AA measurement by LC-MS

To quantify the change in DGLA and AA, one ml of cell suspension was mixed with 1.55 ml water and 0.45 ml methanol, followed by 5 μl of internal standards (DGLA-d6 and AA-d8 (Cayman chemicals, MI, USA)) were added. The samples were then vortexed and centrifuged. The supernatant was collected, and the pH of the supernatant was adjusted to 3 by using HCl. Then, solid-phase extraction was performed by using previously activated SPE (SampliQ Silica C18 ODS, Agilent, CA, USA) by water and methanol. After that, DGLA and AA were eluted out using 2 ml ethyl acetate and the eluted solution was evaporated using vacufuge. After complete drying, the samples were reconstituted with 100 μl ethanol and subjected to LC-MS (Agilent 6300 series MS ion trap and Agilent 1200 series HPLC system) for analysis^16,17^.

### Iminodibenzyl quantification

To quantify the Iminodibenzyl from tumor tissue and organ, the fine powder of tumor tissue and organ was mixed with 3 ml of ethanol and vortexed for 30 s. After vortex, the tumor/organ suspension was kept on ice for one hour. Followed by incubation, the solution was centrifuged, and the supernatant was collected. The extraction procedure was repeated twice and the combined supernatants were dried under vacuum. The extracted Iminodibenzyl was resuspended in 100 μl ethanol and subjected to HPLC analysis by using an established method using 10 mM ammonium acetate buffer (pH adjusted to 2.21 with glacial acetic acid)–methanol (50 + 50, v/v) as a mobile phase, 37° C column temperature, and C18 zorbax column. Iminodibenzyl from the samples was quantified from developed Iminodibenzyl dose *vs*. area under the curve^59^.

### HDAC activity analysis

HDAC activity was then analyzed as per the standard procedure mentioned in the HDAC activity colorimetric assay kit from BioVision (CA, USA). Briefly, protein from different treatment groups was extracted from cells and tumor tissue by using RIPA lysis buffer (Thermo Fisher Scientific, catalog number: 89900) containing protease and phosphatase inhibitor cocktail (Thermo Fisher Scientific, MA, USA). To quantify the percentage HDAC activity reduction, ~50 μg of protein was taken and diluted to 85 μl with ddH_2_O. Then it was mixed with 10 μl of 10× HDAC assay buffer and 5 μl HDAC colorimetric substrate. The plate was then kept on the shaker for 1–2 min, and the plate was incubated at 37° C for one hour to deacetylate the substrate. Followed by incubation, the deacetylation reaction was stopped by adding 10 μl lysine developer (to produce chromophore), and the plate was again incubated for 30 min at 37° C. After 30 min, the reading was observed at 405 nm and calculation was done by considering the Vehicle group at 100%^17^.

### Western analysis

The 4T1 and MDA-MB-231 cells, and tumor harvested from animals treated with Vehicle, DGLA, Iminodibenzyl, and a combination of Iminodibenzyl and DGLA was collected. To validate caspase dependent apoptosis mechanism, cells were collected after pretreatment with 30 μM Z-VAD-FMK followed by combination treatment. Protein was extracted by using RIPA lysis buffer premixed with protease and phosphatase inhibitor cocktail and quantified using BCA protein assay. The proteins were separated by using 4–15% (v/v) TGX gels (Bio-Rad) and immunoblotted on a polyvinylidene difluoride (PVDF) membrane. The membrane was then blocked by using 5% bovine serum albumin (BSA) and incubated with primary antibodies acquired from Cell Signaling Technology (CST) (MA, USA), Abcam (MA, USA), and Santa Cruz Biotechnology (TX, USA). The used primary antibodies were E-Cadherin (ab1416, 1:1000), MMP-2 (SC-13594, 1:200), BCl_2_ (2872S, 1:1000), procaspase-9, procaspase-3, cleaved caspase-3 (ab2302, 1:1000), PARP (9542S, 1:1000), cleaved PARP (ab32064, 1:1000), Paxillin (12065S, 1:1000), Vinculin (SC-73614, 1:200), Snail (SC-271977, 1:200), FAK (3285S, 1:1000), AcH_3_ (K9) (9649S, 1:1000), Total H3 (SC-517576, 1:200), β-Catenin (8480S, 1:1000), Vimentin (V5255, 1:1000) and β-actin (4970S, 1:1000 and G043, 1:1000). The goat anti-Rabbit (IRDye 800 CW, 926-32211, 1:10000) and anti-Mouse (IRDye 680 CW, 926-68070, 1:10000) obtained from LI-COR Biosciences (NE, USA) were used as a secondary antibody. Protein signal was captured by the Li-Cor Odyssey XL System. All blots derived from the same experiment and were processed in parallel. The relative protein intensity was analyzed by Image Studio v.5.2 software^16,17^.

### Immunocytochemistry analysis

Immunocytochemistry study was conducted to analyze the effect of different treatments (Vehicle, 100 μM DGLA, 10 μM Iminodibenzyl, and a combination of 10 μM Iminodibenzyl + 100 μM DGLA) on Cleaved caspase-3, F/G actin, Paxillin, Vinculin, and β-Catenin. After treating with different treatments, the cells were fixed by PFA and permeabilized. Blocking was done by incubating with 10% BSA. The cells were incubated overnight with primary antibodies at 4° C and then with corresponding secondary fluorescent antibodies. Actin filaments were stained by Phalloidin iFlour 488 reagents (Thermo Fisher, catalog number: 12379). The nucleus was stained by DAPI from Electron Microscopy Sciences (PA, USA). The signals were detected by using Carl Zeiss LSM900 confocal microscopy with Airyscan 2^17^.

### Immunohistochemistry analysis

The tumor tissue obtained from the different treatment group were fixed in 4% PFA and tissues were made into the paraffin section as established protocol. The sections were then deparaffinized by xylene and ethanol. Antigen retrieval was conducted by boiling tissue sections in the sodium citrate buffer. The tissue section was then permeabilized using a buffer containing 0.4% v/v Triton X-100 and 1% v/v serum. The tissue section was then incubated with primary and corresponding fluorescent secondary antibodies. The immunofluorescent intensity of the Ki-67, C-PARP, E-Cadherin MMP-2, D5D, and Vimentin was analyzed using Carl Zeiss LSM900 confocal microscopy with Airyscan 2^17^.

### Hematoxylin and eosin (H&E) staining

H&E staining for vital organs from a different group of animals was done at Advanced Imaging & Microscopy facility at North Dakota State University (NDSU). The pathological abnormality was determined by observing the images of tissues under an inverted microscope (Leica Microsystems Model DMi8) in the bright field mode^17^.

### Statistical analysis

GraphPad Prism 9 software was used to perform statistical analyses. The complete randomized design was applied for grouping in vivo mouse study. The data were presented as means ± standard error of the mean (SEM). Data were analyzed by multiple comparisons test using one-way or two-way ANOVA followed by Bonferroni analysis. Statistical significance was indicated by differences with a minimum of *P* *<* 0.05.

### Reporting summary

Further information on research design is available in the Nature Research Reporting Summary linked to this article.

## Supplementary information

Supplementary Information

Reporting summary

## Data Availability

The data supporting the findings of the study will be available upon reasonable request to the corresponding author.
