# Supplementary material for: Iminodibenzyl induced redirected COX-2 activity inhibits breast cancer progression
Source: NPJ Breast Cancer. 2021 Sep 17;7:122. doi: 10.1038/s41523-021-00330-9 (PMC8448825; doi:10.1038/s41523-021-00330-9)

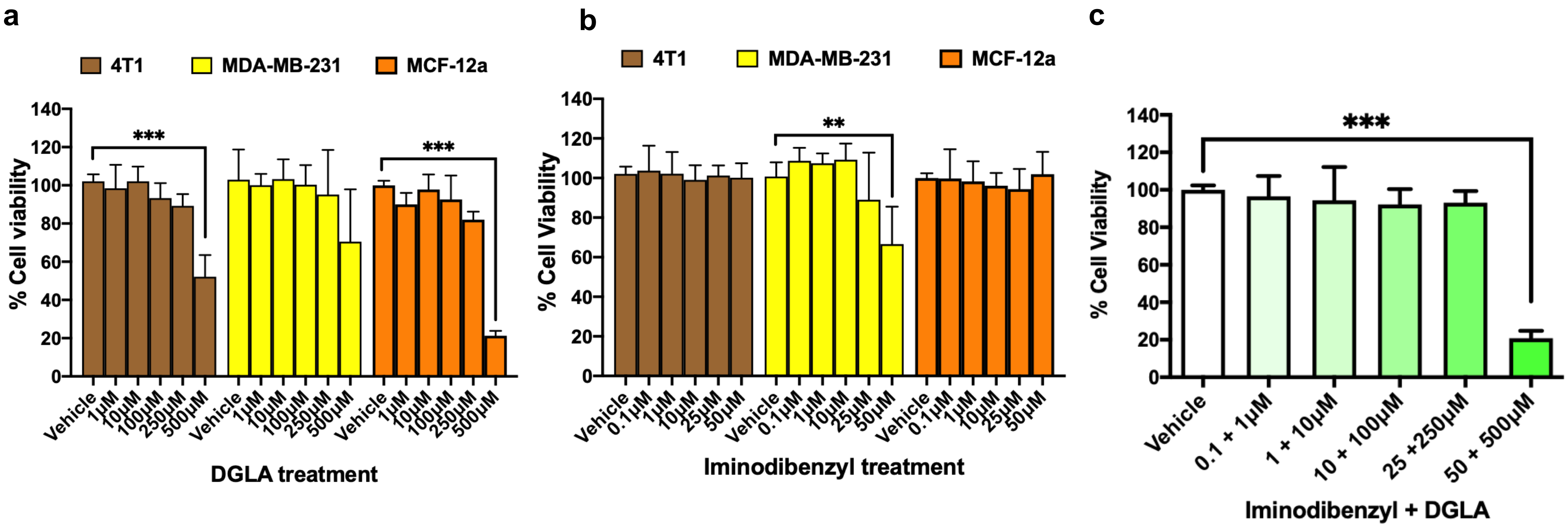


**Supplementary Figure 1: Cell viability analysis after treating cells with DGLA, Iminodibenzyl, and combination of Iminodibenzyl and DGLA.** Cell viability analysis by MTT after 48 hours treatment with gradient doses of **a)** DGLA and **b)** Iminodibenzyl in 4T1, MDA-MB-231, and MCF-12a cell lines. **c)** Cell viability analysis by MTT after 48 hours treatment with different combination doses of Iminodibenzyl and DGLA in MCF-12a cell line. Data represented as mean ± SEM for n=6-10. $^{***}P$<0.001, $^{**}P$<0.01 *vs* vehicle group.

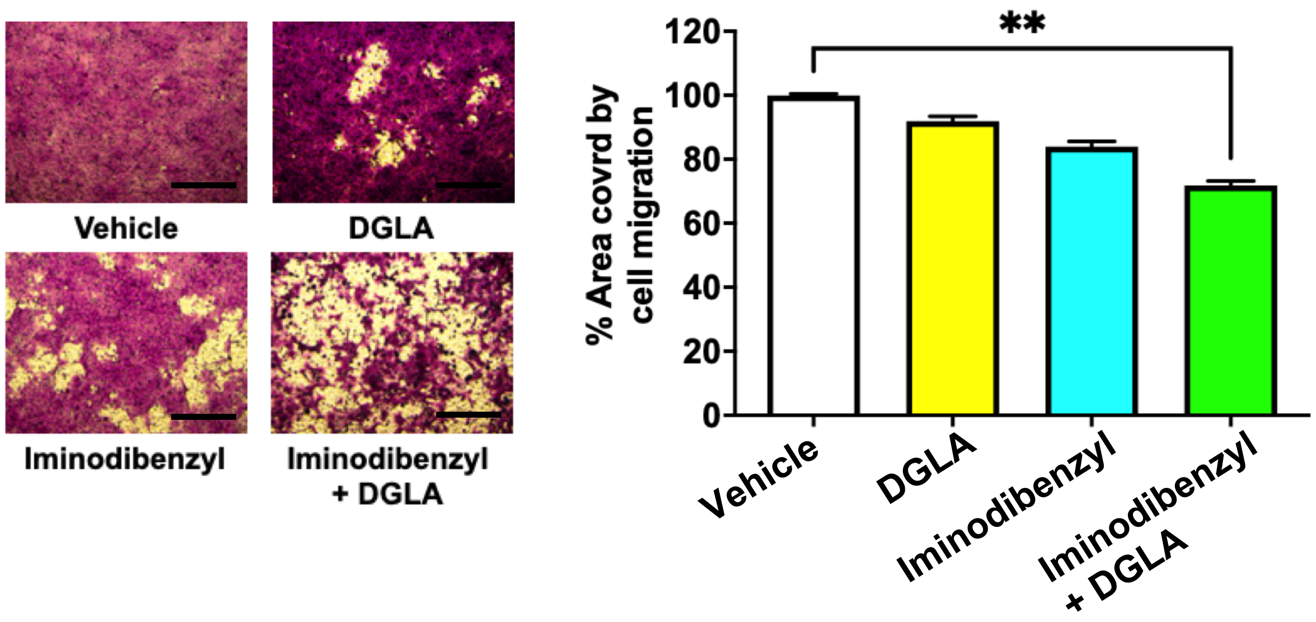


**Supplementary Figure 2: Transwell cell migration analysis depicting decreased 4T1 cell migration after 48 hours treatment with combination of 10μM Iminodibenzyl and 100μM DGLA.** The images were captured by using Leica Microsystems Model DMi8 under 10x**.** Data represented as mean ± SEM for n=3. $^{**}$*P*<0.01 *vs* vehicle group.

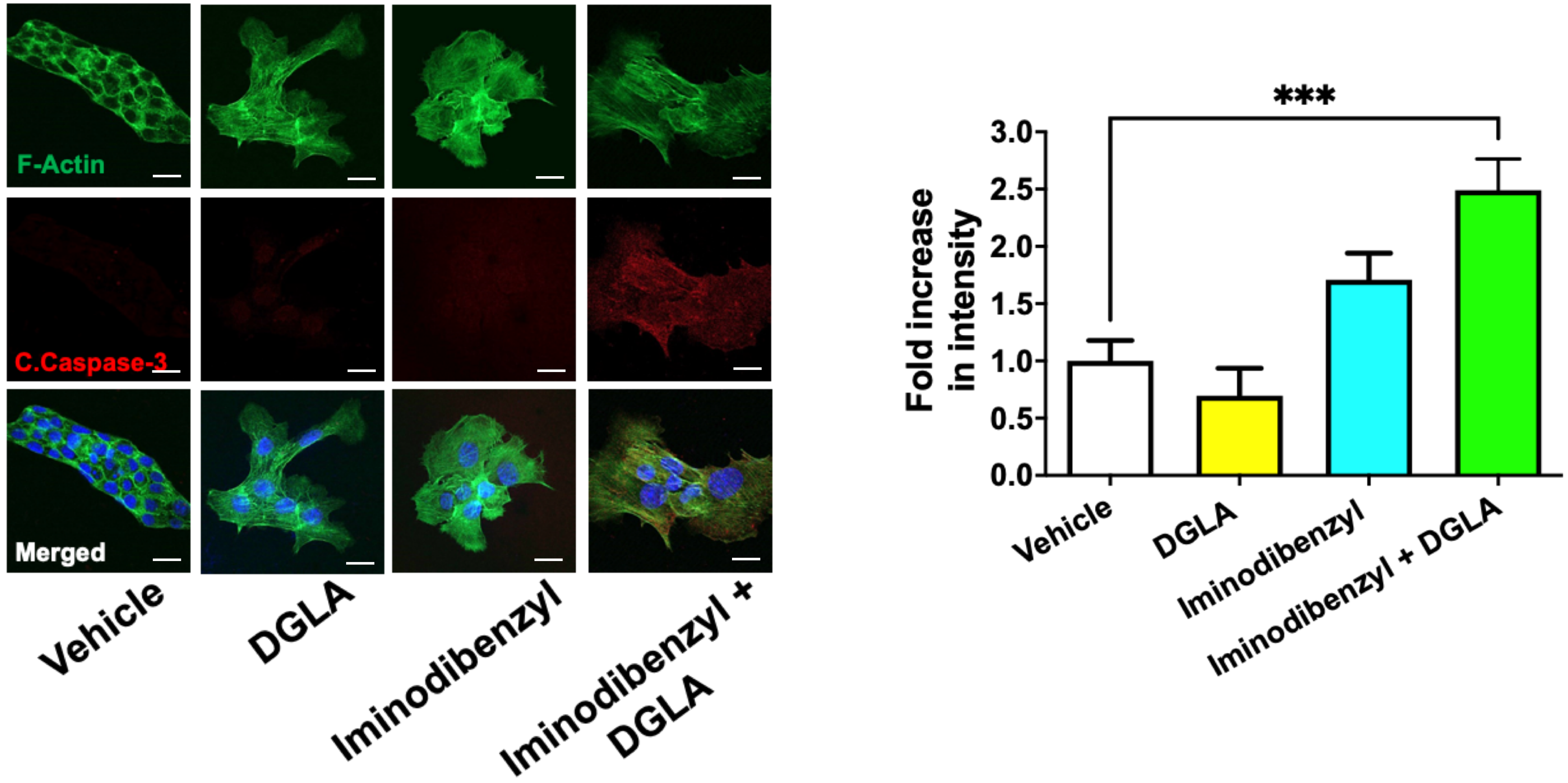


**Supplementary Figure 3: Increased C. Caspase-3 level in 4T1 cells after 48 hours treatment with combination of 10μM Iminodibenzyl and 100μM DGLA.** Scale bar represents 20μm. Data represented as mean ± SEM for n=3. $^{***}P<0.001$ *vs* vehicle group.

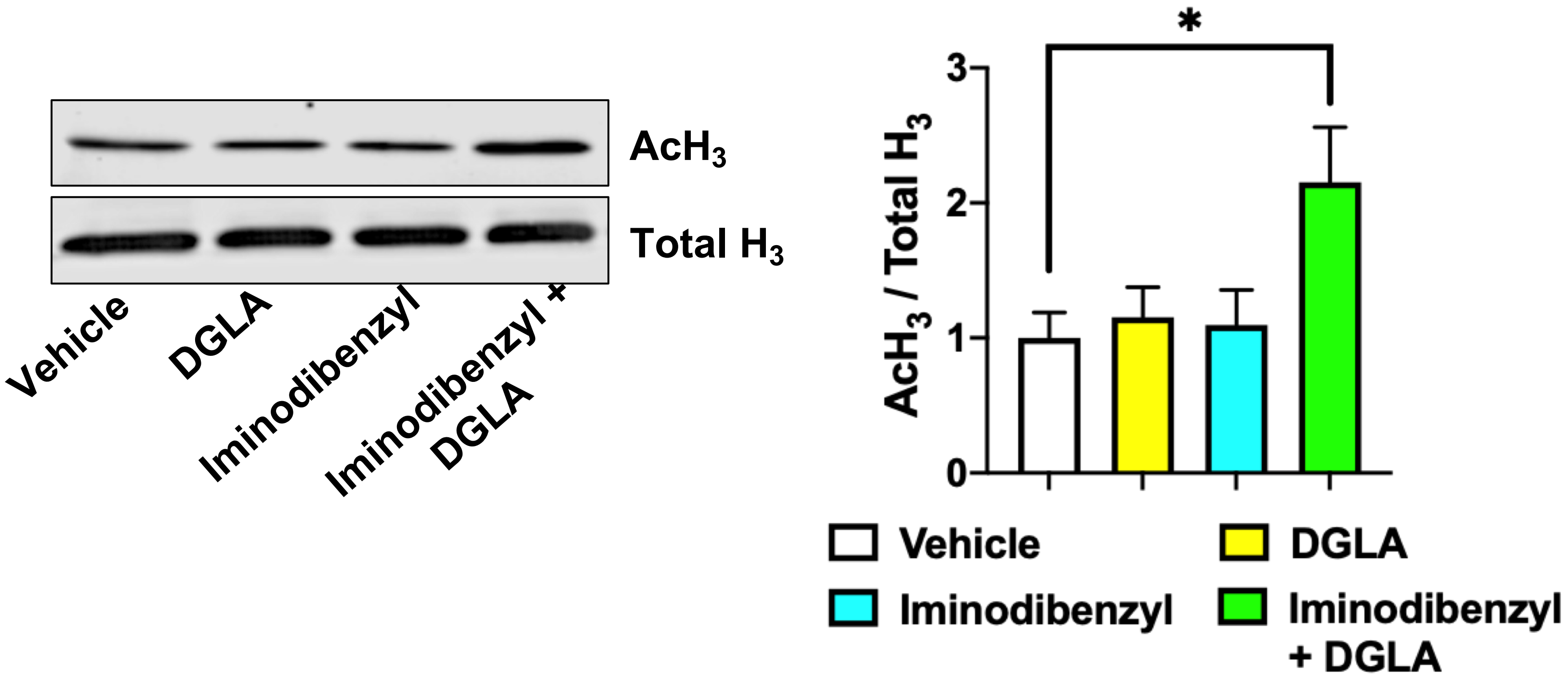


**Supplementary Figure 4: Increased AcH3/Total H3 on providing 10μM Iminodibenzyl + 100μM DGLA as a treatment in MDA-MB-231 cells.** Data represented as mean ± SEM for n=3. *$P<0.05$ *vs* vehicle group.

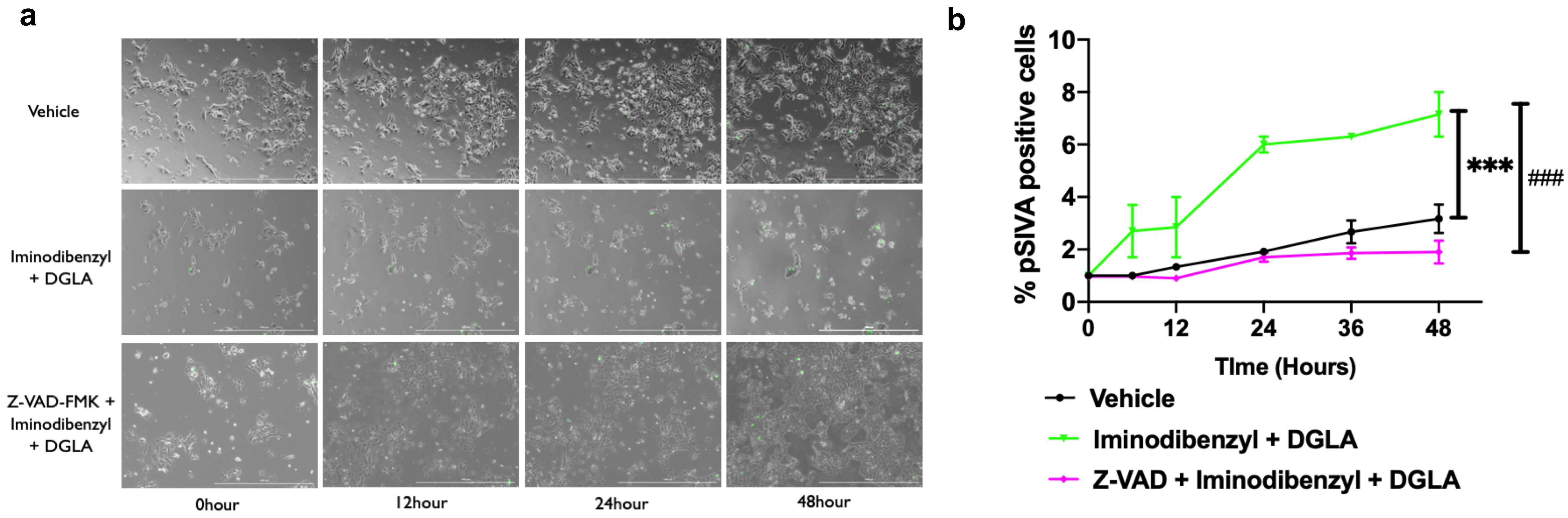


**Supplementary Figure 5: Real-time live 4T1 cell imaging of apoptosis.** (**A**) 4T1 cells were treated with Vehicle, 10μM Iminodibenzyl + 100μM DGLA, and 30μM Z-VAD + 10μM Iminodibenzyl + 100μM DGLA. During 48 h of treatment, cells were incubated with culture medium (37°C, 5% $CO_2$) mixed with Polarity Sensitive Indicator of Viability & Apoptosis (pSIVA) and propidium iodide (ab129817) for imaging under time-lapse through Lionheart FX Automated Microscope microscopy over time under 10x. Green fluorescence indicated pSIVA positive cells. (b) The percentage of pSIVA positive cells was quantified at different time points at the same field. Data represented as mean ± SEM for n=3. $^{***}$*P*<0.001 *vs* vehicle group, $^{\#\#\#}$*p<0.001 vs.* Iminodibenzyl + DGLA group at 48 h.

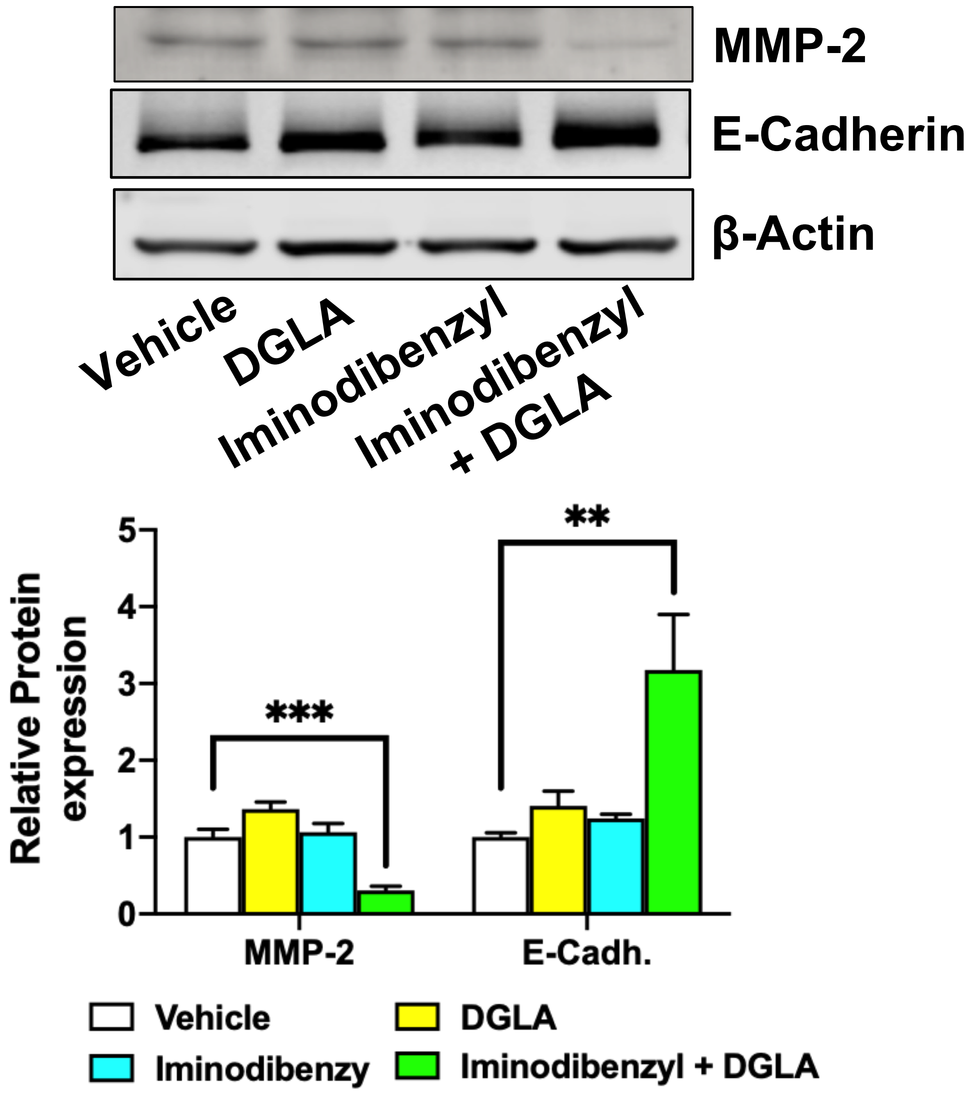


**Supplementary Figure 6: Protective effect of Iminodibenzyl and DGLA combination treatment on EMT markers in MDA-MB-231 cells.** Data represented as mean ± SEM for n=3. $^{**}P<0.01$, ***P<0.001 *vs* vehicle group.

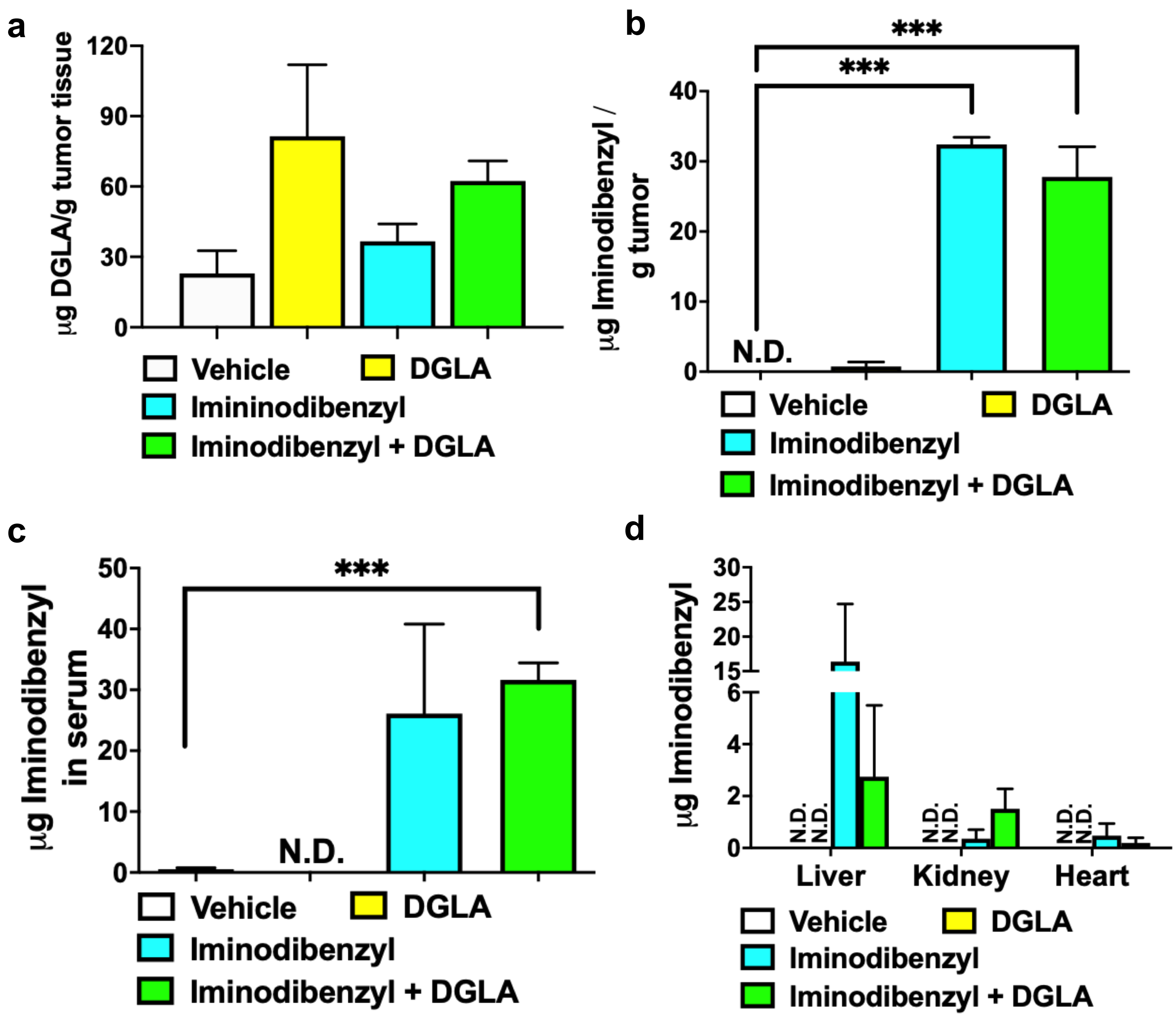


**Supplementary Figure 7: DGLA and Iminodibenzyl content analysis after different treatment. a** DGLA quantification in tumors obtained from animals treated with different treatment. Iminodibenzyl quantification in tumors (**b**), serum (**c**), and different organs (**d**) obtained from animals treated with Vehicle, DGLA, Iminodibenzyl, and combination of DGLA and Iminodibenzyl. N=3. *** *P<0.001 vs.* vehicle and N.D.: Not Detectable.

**Fig. 3a**

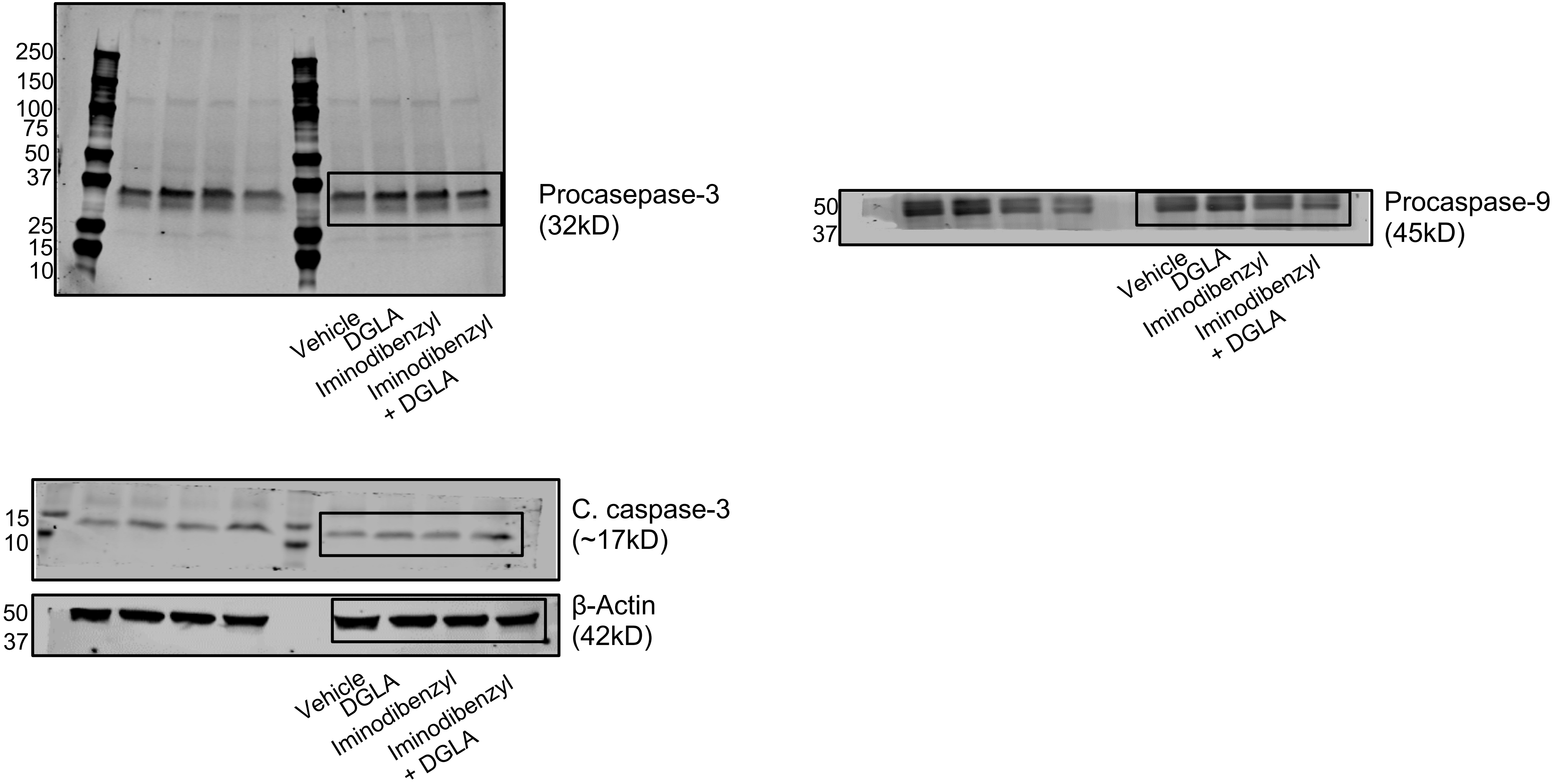
250
150
100
75
50
37
25
15
10
Procasepase-3
(32kD)
Vehicle
DGLA
Iminodibenzyl
Iminodibenzyl
+ DGLA
50
37
Procaspase-9
(45kD)
Vehicle
DGLA
Iminodibenzyl
Iminodibenzyl
+ DGLA
15
10
C. caspase-3
(~17kD)
50
37
β-Actin
(42kD)
Vehicle
DGLA
Iminodibenzyl
Iminodibenzyl
+ DGLA

**Fig. 3b**

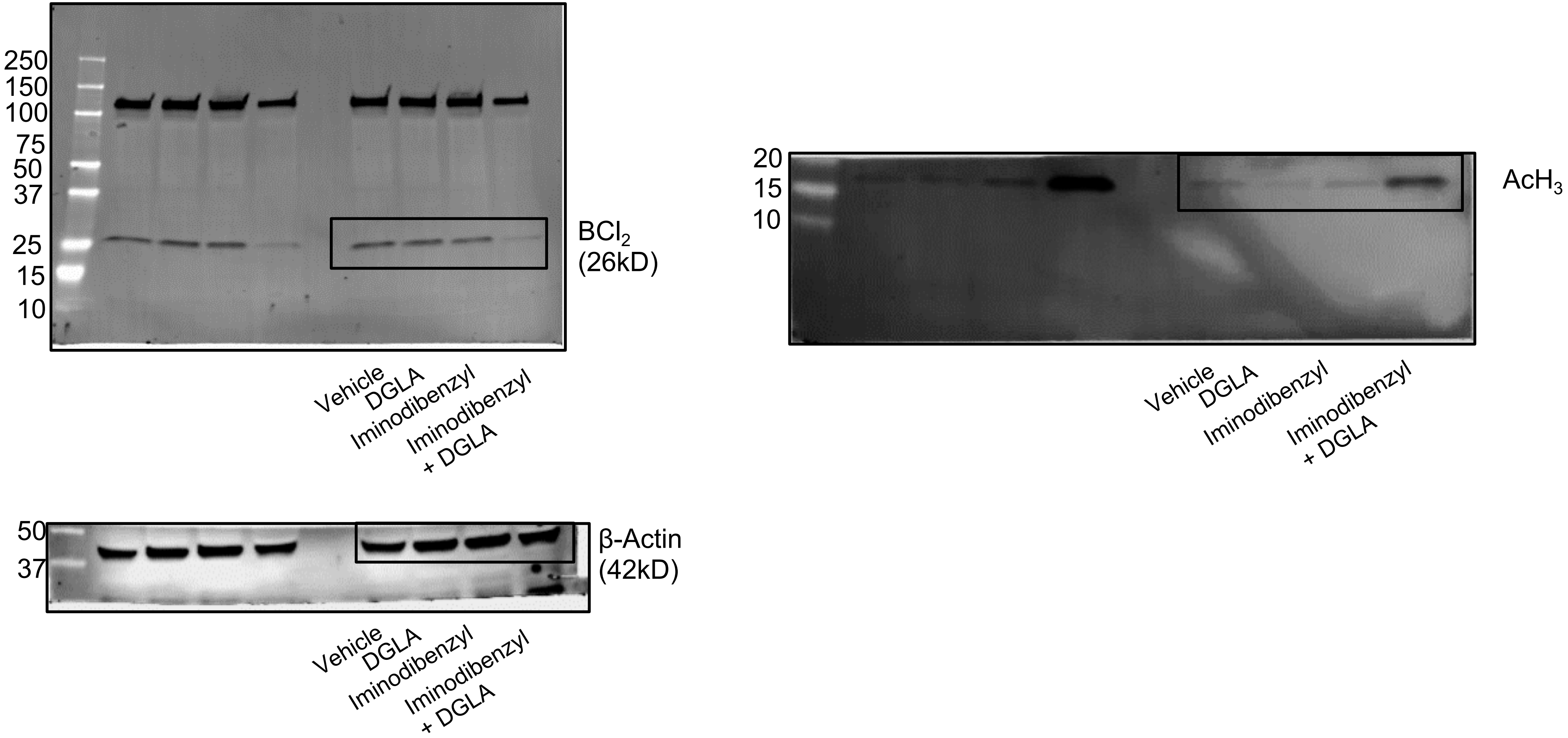
250
150
100
75
50
37
25
15
10
BCl2 (26kD)
Vehicle
DGLA
Iminodibenzyl
Iminodibenzyl + DGLA
20
15
10
AcH3
Vehicle
DGLA
Iminodibenzyl
Iminodibenzyl + DGLA
50
37
β-Actin (42kD)
Vehicle
DGLA
Iminodibenzyl
Iminodibenzyl + DGLA

**Fig. 3c**

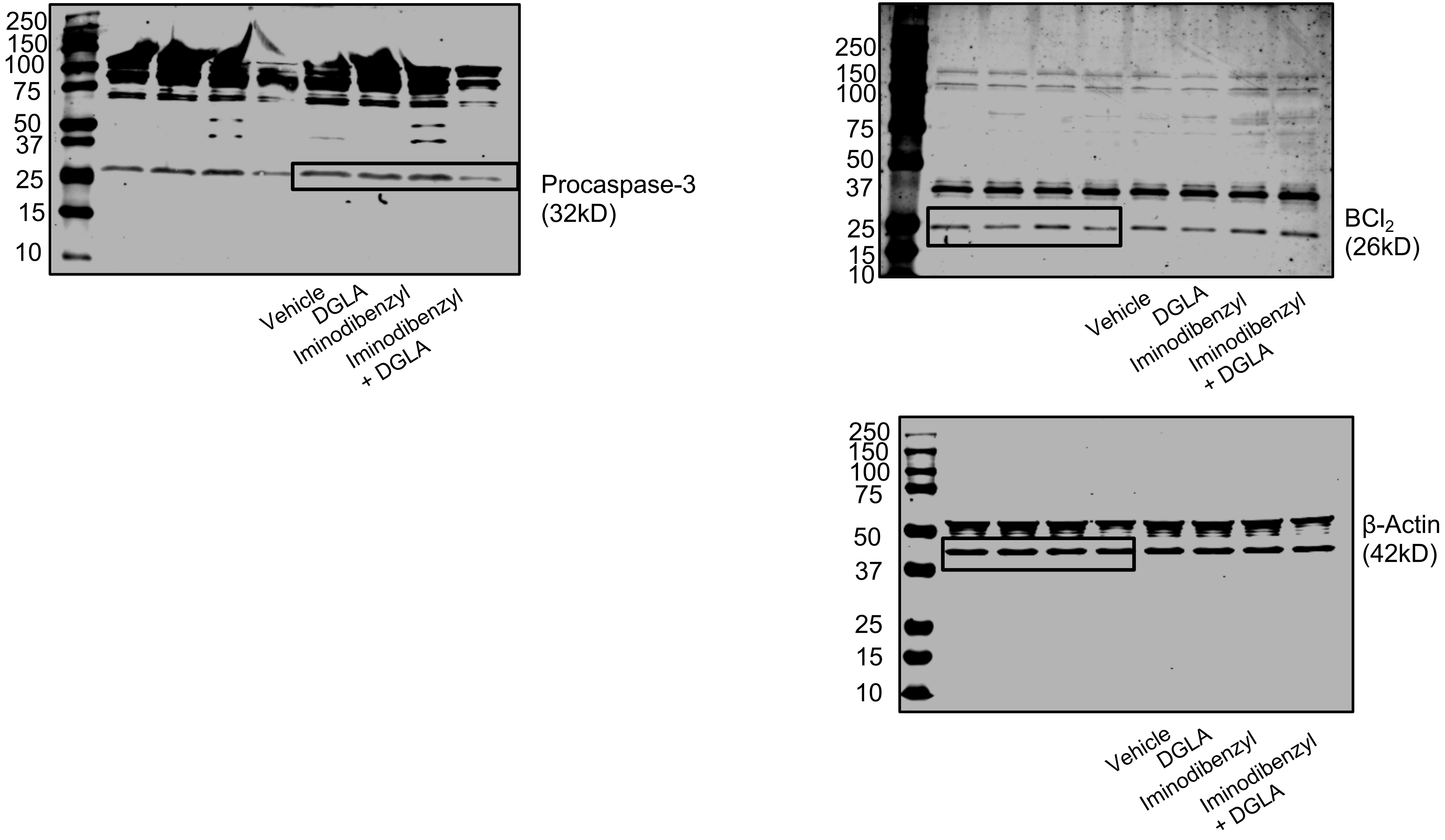
250
150
100
75
50
37
25
15
10
Procaspase-3
(32kD)
Vehicle
DGLA
Iminodibenzyl
Iminodibenzyl
+ DGLA
250
150
100
75
50
37
25
15
10
$BCl_2$
(26kD)
Vehicle
DGLA
Iminodibenzyl
Iminodibenzyl
+ DGLA
250
150
100
75
50
37
25
15
10
β-Actin
(42kD)
Vehicle
DGLA
Iminodibenzyl
Iminodibenzyl
+ DGLA

**Fig. 4c**

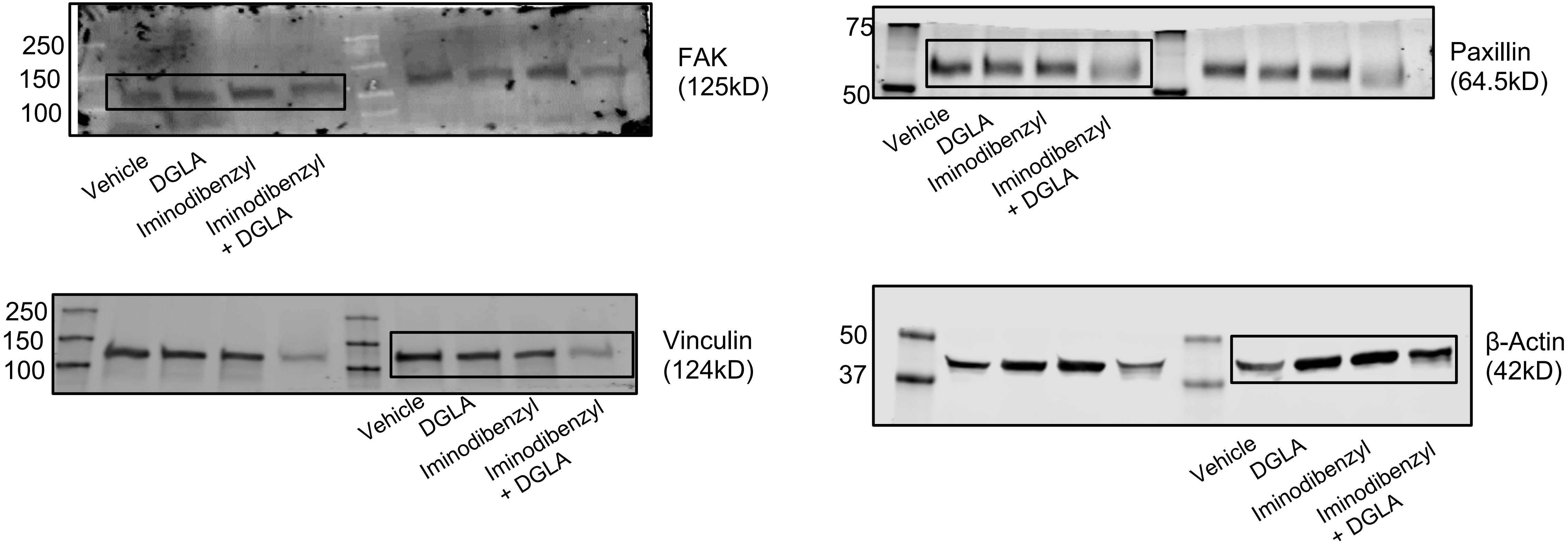

250
150
100
FAK
(125kD)
Vehicle
DGLA
Iminodibenzyl
Iminodibenzyl + DGLA
75
50
Paxillin
(64.5kD)
Vehicle
DGLA
Iminodibenzyl
Iminodibenzyl + DGLA
250
150
100
Vinculin
(124kD)
Vehicle
DGLA
Iminodibenzyl
Iminodibenzyl + DGLA
50
37
β-Actin
(42kD)
Vehicle
DGLA
Iminodibenzyl
Iminodibenzyl + DGLA

**Fig. 4e**

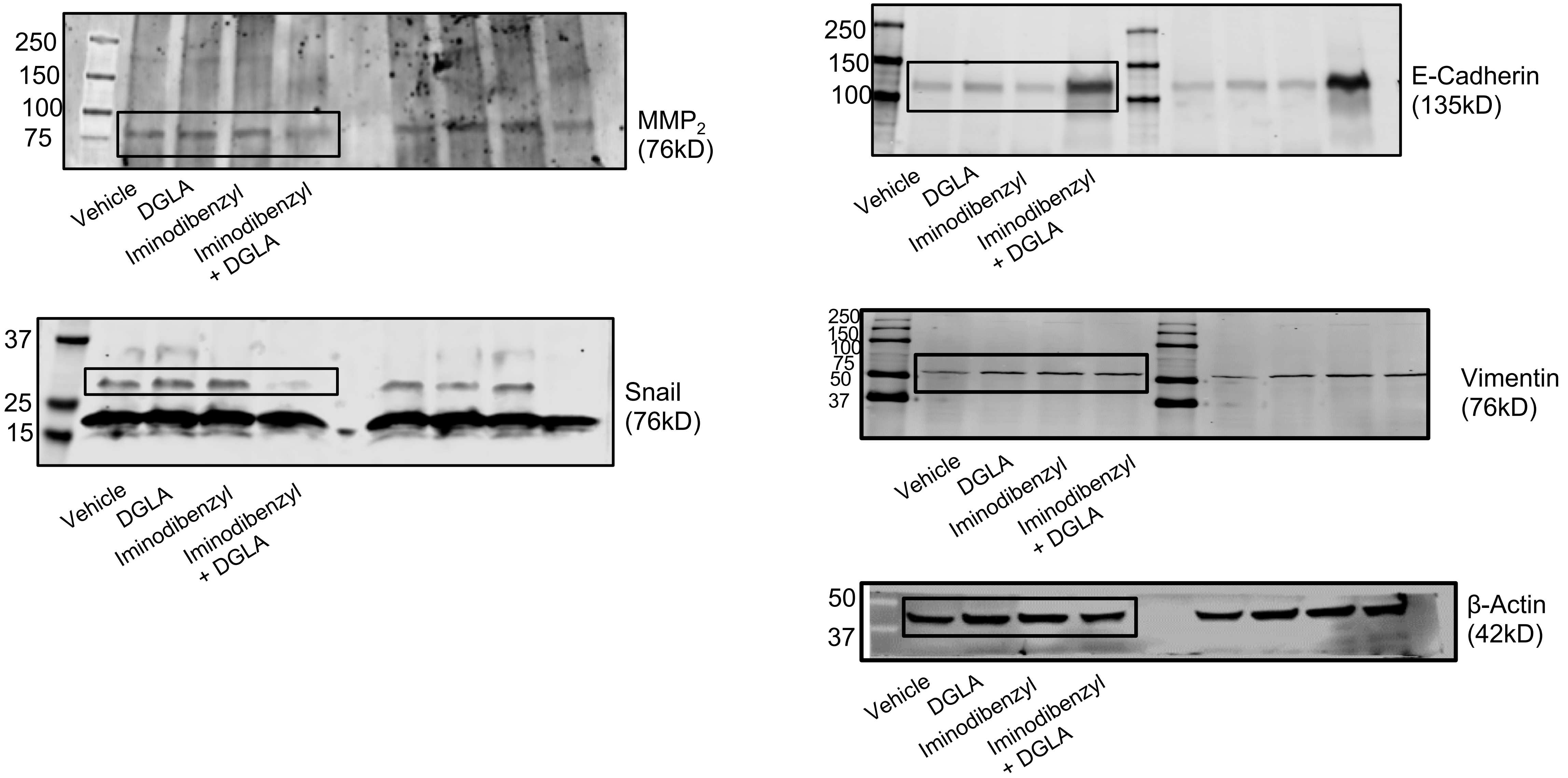
250
150
100
75
MMP2
(76kD)
Vehicle
DGLA
Iminodibenzyl
Iminodibenzyl + DGLA
37
25
15
Snail
(76kD)
Vehicle
DGLA
Iminodibenzyl
Iminodibenzyl + DGLA
250
150
100
E-Cadherin
(135kD)
Vehicle
DGLA
Iminodibenzyl
Iminodibenzyl + DGLA
250
150
100
75
50
37
Vimentin
(76kD)
Vehicle
DGLA
Iminodibenzyl
Iminodibenzyl + DGLA
50
37
β-Actin
(42kD)
Vehicle
DGLA
Iminodibenzyl
Iminodibenzyl + DGLA

**Fig. 4g**

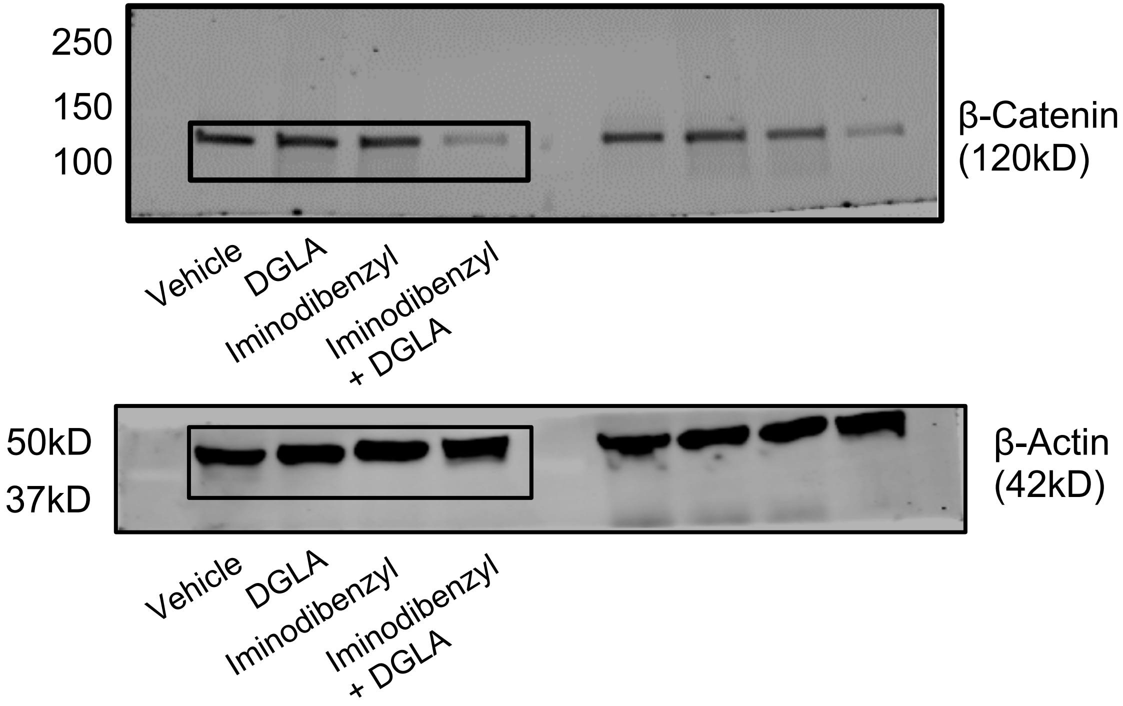
250
150
100
β-Catenin
(120kD)
Vehicle
DGLA
Iminodibenzyl
Iminodibenzyl + DGLA
50kD
37kD
β-Actin
(42kD)
Vehicle
DGLA
Iminodibenzyl
Iminodibenzyl + DGLA

**Fig. 6b**

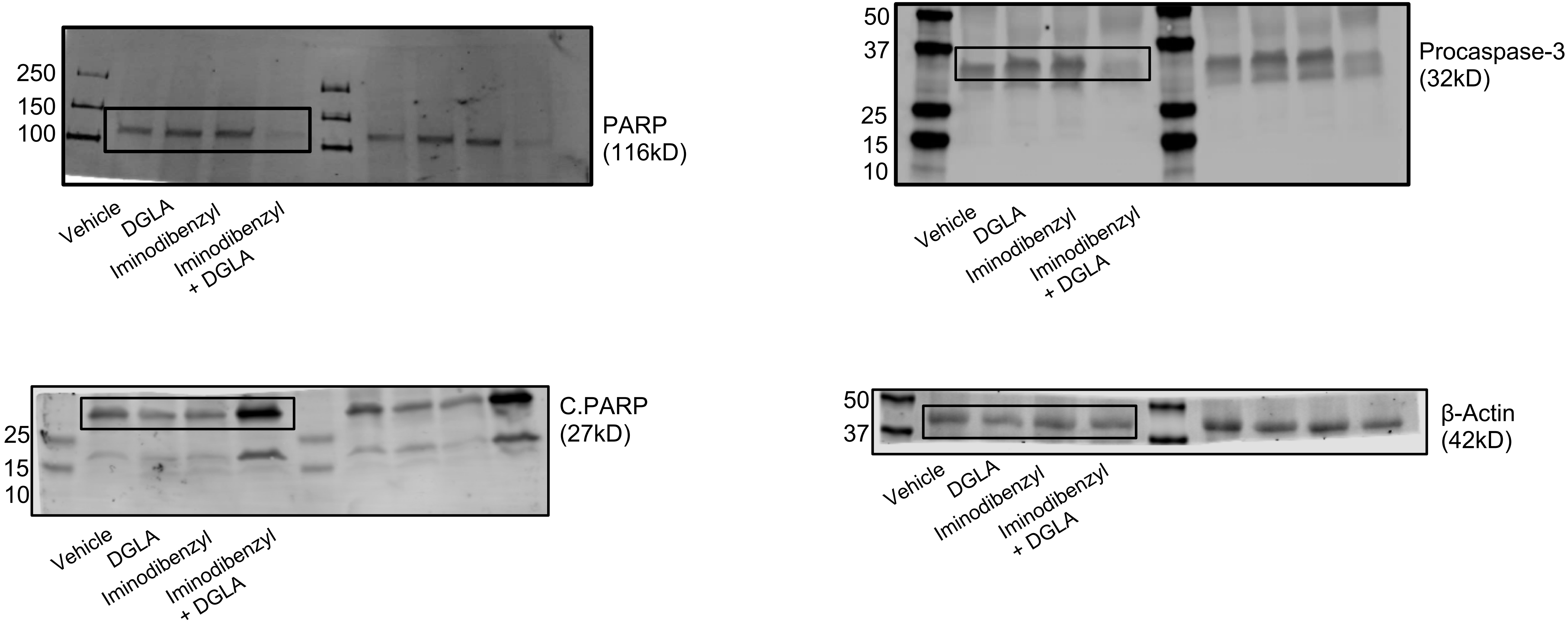

250
150
100
PARP
(116kD)
Vehicle
DGLA
Iminodibenzyl
Iminodibenzyl + DGLA
50
37
25
15
10
Procaspase-3
(32kD)
Vehicle
DGLA
Iminodibenzyl
Iminodibenzyl + DGLA
25
15
10
C.PARP
(27kD)
Vehicle
DGLA
Iminodibenzyl
Iminodibenzyl + DGLA
50
37
β-Actin
(42kD)
Vehicle
DGLA
Iminodibenzyl
Iminodibenzyl + DGLA

**Fig. 6c**

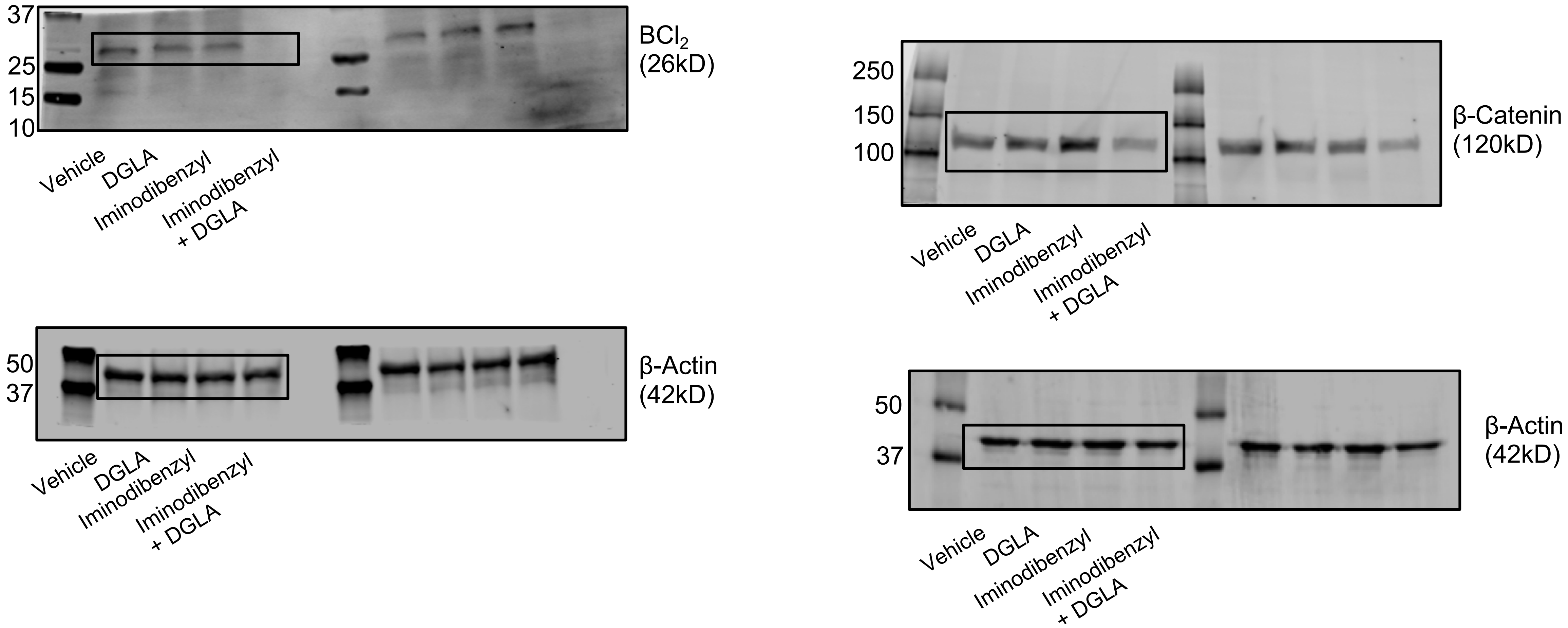
37
25
15
10
$BCl_2$
(26kD)
Vehicle
DGLA
Iminodibenzyl
Iminodibenzyl + DGLA
250
150
100
β-Catenin
(120kD)
Vehicle
DGLA
Iminodibenzyl
Iminodibenzyl + DGLA
50
37
β-Actin
(42kD)
Vehicle
DGLA
Iminodibenzyl
Iminodibenzyl + DGLA
50
37
β-Actin
(42kD)
Vehicle
DGLA
Iminodibenzyl
Iminodibenzyl + DGLA

# Supplementary Figure 4

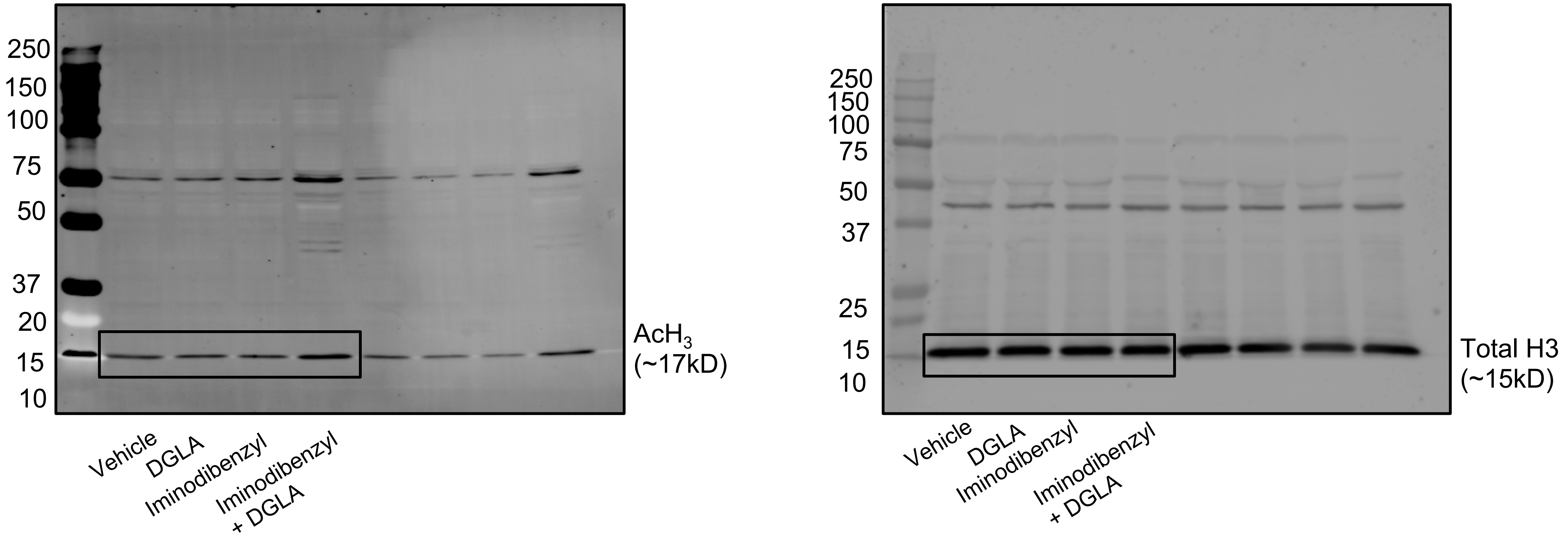

# Supplementary Figure 6

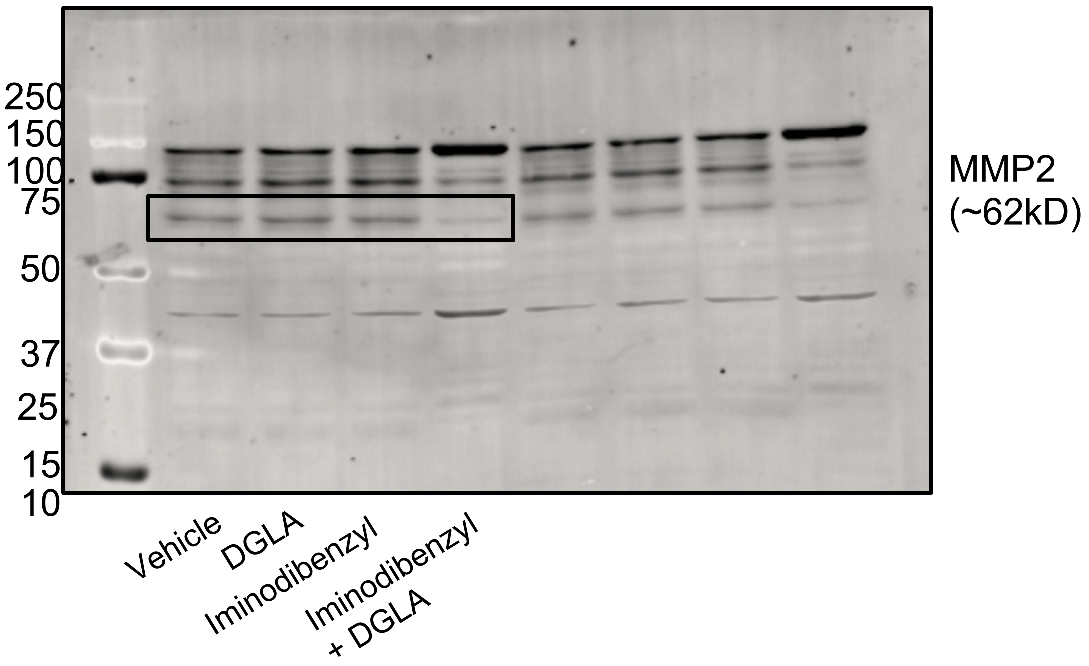


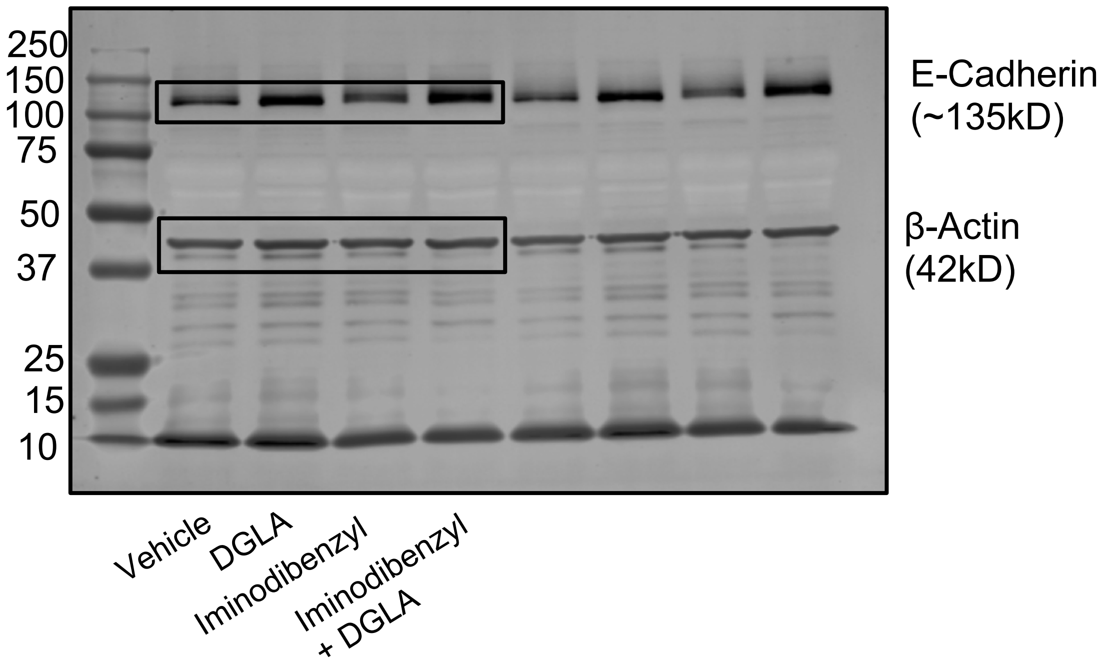

Supplement: Supplementary file 1 — Supplementary Information [file 41523_2021_330_MOESM1_ESM.pdf]
