# Supplementary material for: Iminodibenzyl induced redirected COX-2 activity inhibits breast cancer progression
Source: NPJ Breast Cancer. 2021 Sep 17;7:122. doi: 10.1038/s41523-021-00330-9 (PMC8448825; doi:10.1038/s41523-021-00330-9)
Supplement: Supplementary file 2 — Reporting summary [file 41523_2021_330_MOESM2_ESM.pdf]

nature portfolio

Corresponding author(s): Venkatachalem Sathish

Last updated by author(s): Aug 16, 2021

Reporting Summary

Nature Portfolio wishes to improve the reproducibility of the work that we publish. This form provides structure for consistency and transparency in reporting. For further information on Nature Portfolio policies, see our Editorial Policies and the Editorial Policy Checklist.

Statistics

For all statistical analyses, confirm that the following items are present in the figure legend, table legend, main text, or Methods section.

n/a	Confirmed	
☐	☒	The exact sample size (*n*) for each experimental group/condition, given as a discrete number and unit of measurement
☐	☒	A statement on whether measurements were taken from distinct samples or whether the same sample was measured repeatedly
☐	☒	The statistical test(s) used AND whether they are one- or two-sided<br>*Only common tests should be described solely by name; describe more complex techniques in the Methods section.*
☒	☐	A description of all covariates tested
☒	☐	A description of any assumptions or corrections, such as tests of normality and adjustment for multiple comparisons
☐	☒	A full description of the statistical parameters including central tendency (e.g. means) or other basic estimates (e.g. regression coefficient) AND variation (e.g. standard deviation) or associated estimates of uncertainty (e.g. confidence intervals)
☐	☒	For null hypothesis testing, the test statistic (e.g. *F*, *t*, *r*) with confidence intervals, effect sizes, degrees of freedom and *P* value noted<br>*Give P values as exact values whenever suitable.*
☒	☐	For Bayesian analysis, information on the choice of priors and Markov chain Monte Carlo settings
☒	☐	For hierarchical and complex designs, identification of the appropriate level for tests and full reporting of outcomes
☒	☐	Estimates of effect sizes (e.g. Cohen's *d*, Pearson's *r*), indicating how they were calculated

Our web collection on statistics for biologists contains articles on many of the points above.

Software and code

Policy information about availability of computer code

Data collection	The images for immunofluorescence and IHC was acquired by using Zeiss LSM900 with airyscan 2 microscope. The images for in brightfield mode was acquired by using Leica DMi8. Live cell imaging microscopy study was done by LionHeart FX.
Data analysis	Data analysis was done by ImageJ and statistical analysis was done by using GraphPad Prism 9.

For manuscripts utilizing custom algorithms or software that are central to the research but not yet described in published literature, software must be made available to editors and reviewers. We strongly encourage code deposition in a community repository (e.g. GitHub). See the Nature Portfolio guidelines for submitting code & software for further information.

Data

Policy information about availability of data

All manuscripts must include a data availability statement. This statement should provide the following information, where applicable:

- Accession codes, unique identifiers, or web links for publicly available datasets
- A description of any restrictions on data availability
- For clinical datasets or third party data, please ensure that the statement adheres to our policy

All data are available upon request.

Field-specific reporting

Please select the one below that is the best fit for your research. If you are not sure, read the appropriate sections before making your selection.

☒ Life sciences ☐ Behavioural & social sciences ☐ Ecological, evolutionary & environmental sciences

For a reference copy of the document with all sections, see nature.com/documents/nr-reporting-summary-flat.pdf

Life sciences study design

All studies must disclose on these points even when the disclosure is negative.

Sample size	Based on our previous studies, the sample size for animal study was determined.
Data exclusions	*Describe any data exclusions. If no data were excluded from the analyses, state so OR if data were excluded, describe the exclusions and the rationale behind them, indicating whether exclusion criteria were pre-established.*
Replication	All experiments have minimum of three replicates.
Randomization	The animals were randomized at the beginning of the treatment.
Blinding	The animal study was non-blinded.

Reporting for specific materials, systems and methods

We require information from authors about some types of materials, experimental systems and methods used in many studies. Here, indicate whether each material, system or method listed is relevant to your study. If you are not sure if a list item applies to your research, read the appropriate section before selecting a response.

Materials & experimental systems

n/a	Involved in the study	
☐	☒	Antibodies
☐	☒	Eukaryotic cell lines
☒	☐	Palaeontology and archaeology
☐	☒	Animals and other organisms
☒	☐	Human research participants
☒	☐	Clinical data
☒	☐	Dual use research of concern

Methods

n/a	Involved in the study	
☒	☐	ChIP-seq
☐	☒	Flow cytometry
☒	☐	MRI-based neuroimaging

Antibodies

Antibodies used

Antibodies from Cell Signaling Technology
AcH3 (9649S)
BCl2 (2872S)
B-Actin (4970S)
PARP (9542S)
MMP-9 (2270S)
FAK (3285S)
B-Catenin (8480S)
Paxillin (12065S)

Antibodies from AbCam
C.PARP (ab32064)
C.Caspase 3 (ab2302)
MMP-2 (ab37150)
E-Cadherin (ab1416)
FADS1 (D5D) (ab126706)
Vimentin (ab8978)
Ki-67 (16667)

Antibodies from SantaCruz Biotechnology
Vinculin (SC-73614)
Snai (SC-271977)
TotalH3 (SC517576)
MMP-2 (SC13594)

	Antibodies from Abm<br>B-Actin (G043)<br><br>Antibody from Sigma Aldrich<br>Vimentin (V5255)
Validation	All antibodies were validated for target specificity by the Vendor.

Eukaryotic cell lines

Policy information about cell lines

Cell line source(s)	MDA-MB-231, 4T1, MCF-7a, MCF-12a
Authentication	All cell lines were purchased from ATCC and authenticated by IDEXX before conducting animal studies for possible contamination
Mycoplasma contamination	All cell lines were confirmed for mycoplasma contamination
Commonly misidentified lines<br>(See ICLAC register)	*Name any commonly misidentified cell lines used in the study and provide a rationale for their use.*

Animals and other organisms

Policy information about studies involving animals; ARRIVE guidelines recommended for reporting animal research

Laboratory animals	The female Nu/J mice for conducting in vivo studies were acquired from the Jax lab.
Wild animals	n/a
Field-collected samples	n/a
Ethics oversight	The animals purchasing and study was approved by the IACUC at NDSU. The in vitro study conducted was approved by IBC at NDSU.

Note that full information on the approval of the study protocol must also be provided in the manuscript.

Flow Cytometry

Plots

Confirm that:

☒ The axis labels state the marker and fluorochrome used (e.g. CD4-FITC).

☒ The axis scales are clearly visible. Include numbers along axes only for bottom left plot of group (a 'group' is an analysis of identical markers).

☐ All plots are contour plots with outliers or pseudocolor plots.

☒ A numerical value for number of cells or percentage (with statistics) is provided.

Methodology

Sample preparation	The cells were collected by trypsinization after the treatment period and washed twice with cold PBS and then resuspend cells in 1X Binding Buffer at a concentration of ~1 x 10^6 cells/ml.<br>Then, the cells (~1 x 10^5 cells) were transferred to a 5 ml flow tube.<br>The solution was mixed with 5ul PI and 5ul Annexin V FITC.<br>The cells were gently mixed and incubated for 30 min at room temperature in dark.<br>400 µl of 1X Binding Buffer was added to each tube and the reading was taken within one hour.
Instrument	Acuri C6
Software	Acuri C6
Cell population abundance	A minimum cell count of 10,000 was used.
Gating strategy	The cells were gated as mentioned in the figures. The standard templet for PI-Annexin V analysis is developed in house for data capturing and analysis.

☐ Tick this box to confirm that a figure exemplifying the gating strategy is provided in the Supplementary Information.
